# Untargeted Gut Metabolomics to Delve the Interplay between Selenium Supplementation and Gut Microbiota

**DOI:** 10.1021/acs.jproteome.1c00411

**Published:** 2021-11-04

**Authors:** Belén Callejón-Leblic, Marta Selma-Royo, María Carmen Collado, José Luis Gómez-Ariza, Nieves Abril, Tamara García-Barrera

**Affiliations:** † Research Center of Natural Resources, Health and the Environment (RENSMA), Department of Chemistry, Faculty of Experimental Sciences, Campus El Carmen, University of Huelva, Fuerzas Armadas Avenue, 21007 Huelva, Spain; ‡ Institute of Agrochemistry and Food Technology−National Research Council (IATA-CSIC), Department of Biotechnology, Agustín Escardino 7, 46980 Paterna, Valencia, Spain; § Department of Biochemistry and Molecular Biology, University of Córdoba, Campus de Rabanales, Edificio Severo Ochoa, 14071 Córdoba, Spain

**Keywords:** selenium, gut metabolomics, gut microbiota, functional food, mass spectrometry, untargeted metabolomics

## Abstract

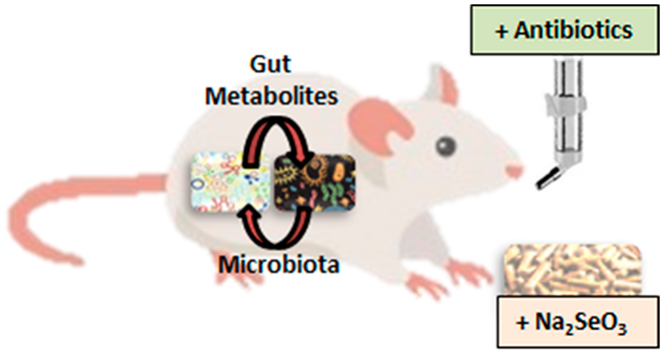

Selenium (Se) is an essential trace element with important health roles due to the antioxidant properties of selenoproteins. To analyze the interplay between Se and gut microbiota, gut metabolomic profiles were determined in conventional (C) and microbiota depleted mice (Abx) after Se-supplementation (Abx-Se) by untargeted metabolomics, using an analytical multiplatform based on GC-MS and UHPLC-QTOF-MS (MassIVE ID MSV000087829). Gut microbiota profiling was performed by 16S rRNA gene amplicon sequencing. Significant differences in the levels of about 70% of the gut metabolites determined, including fatty acyls, glycerolipids, glycerophospholipids, and steroids, were found in Abx-Se compared to Abx, and only 30% were different between Abx-Se and C, suggesting an important effect of Se-supplementation on Abx mice metabolism. At genus level, the correlation analysis showed strong associations between metabolites and gut bacterial profiles. Likewise, higher abundance of *Lactobacillus spp.*, a potentially beneficial genus enriched after Se-supplementation, was associated with higher levels of prenol lipids, phosphatidylglycerols (C-Se), steroids and diterpenoids (Abx-Se), and also with lower levels of fatty acids (Abx-Se). Thus, we observed a crucial interaction between Se intake–microbiota–metabolites, although further studies to clarify the specific mechanisms are needed. This is the first study about untargeted gut metabolomics after microbiota depletion and Se-supplementation.

## 1 Introduction

Selenium (Se) is an oligoelement with antioxidant properties^1^ that can antagonize the action of several xenobiotics.^2^ Se and selenoproteins have chemopreventive properties against cancer^3^ and reduce the incidence and severity of various viral infections including COVID-19.^4^ Due to their relationship with health, functional foods and nutraceuticals enriched with Se^5,6^ have been extensively described. Moreover, the effect of Se on the intestinal microbiota, by shaping the microbial composition and diversity, and also by improving intestinal mucositis, has also been suggested.^7^

Dysbiosis in gut microbiota due environmental factors, diet, or drug use is associated with adverse health outcomes^8,9^ including the development of several disorders, such as metabolic syndrome, obesity, adiposity, type 2 diabetes, dyslipidemia, or cardiovascular diseases.^10−12^ In addition, gut dysbiosis has also been related to alterations in the gut metabolome^13^ due the changes on gut microbiota metabolism. Gut content and fecal samples contain a great number of metabolites that reflect the results of nutrient ingestion, digestion, and absorption by gut microbiota, and consequently have a key impact on host metabolism.^14^ To this end, the integration of gut microbiota taxonomy and gut metabolome can provide interesting information about the mechanisms used in the host–microbiota interactions.

Metabolomics is considered a powerful omic approach that allows determining a wide number of metabolites in a large variety of biological samples including fecal and gut content. The analytical techniques used in the current metabolomic studies are mainly based on nuclear magnetic resonance spectroscopy (NMR), gas chromatography mass spectrometry (GC-MS), and liquid chromatography mass spectrometry coupled to several analyzers, such as quadrupole-time-of-flight (LC-QTOF-MS)^15−17^ or linear trap quadrupole-Orbitrap (LC-LTQ-Orbitrap).^15,18^ Although NMR has several advantages (e.g., fast sample preparation, nondestructive sample technique, high sample throughput), its main drawback is the poor sensitivity that transforms MS into an excellent analytical tool for metabolomics.^19^ Only a few authors have combined more than one analytical technique for gut metabolomics that led to a poor metabolite coverage.^16,18^

Although the role of Se^20,21^ and other dietary minerals^22^ in shaping gut microbiota has been reported, there are not many studies that have described associations between altered gut microbiota and gut metabolites due to Se-supplementation.^23,24^ Under our knowledge, this is the first work describing an untargeted metabolomics approach to analyze gut metabolites after microbiota depletion and/or Se-supplementation and establishing associations between gut metabolites and gut microbiota.

The aim of this study was to understand the impact of Se-supplementation on the gut metabolome profile and the association with the microbiota profiles in conventional (C) and microbiota-depleted mice (Abx). To reach these objectives, we have combined an analytical multiplatform based on GC-MS and ultrahigh performance liquid chromatography (UHPLC) coupled to QTOF-MS for gut metabolomics to cover a wide number of metabolites from gut samples. Gut microbiota composition was determined by using 16S rRNA gene sequencing.

## 2 Materials and Methods

### 2.1 Chemicals and Reagents

Methanol, acetonitrile (LC-MS grade), and formic acid were supplied by Fisher Scientific (Leicestershire, UK). Pyridine, methoxylamine hydrochloride, *N*-methyl-N-(trimethylsilyl) trifluoroacetamide (MSTFA), and the antibiotics ampicillin, neomycin, metronidazole vancomycin, and the antifungal amphotericin B were obtained from Aldrich (Steinheim, Germany). Water was purified with a Milli-Q Gradient system (Millipore, Watford, UK). DNA Purification Kit was obtained from Macherey-Nagel (Duren, Germany), Master-Pure the DNA extraction Kit was obtained from Epicenter (Madison, WI, US), and NextEra Index Kit was obtained from Illumina, (San Diego, CA, US).

### 2.2 Study Design

Animal handling (Figure S1) was performed at the Animal Experimentation Service of the University of Córdoba (SAEX-UCO), by qualified personnel and following European Community animal care guidelines. In addition, the study was approved by the consent of the Ethical Committee of the University of Córdoba (Spain) (Code No. 02-01-2019-001). The study design has been previously described.^25^ Briefly, a total of 40 male *Mus musculus* mice (inbred BALB/c strain, 8 weeks old, 23–25 g of weight) were obtained from Charles River Laboratories (Spain) and were divided into four groups (10 mice per group). Mice in the control group (C) were fed a rodent diet for 3 weeks (0.20 mg Se kg^−1^ chow)(; mice in C-Se group were fed a Se-enriched diet containing 0.65 mg Se kg_^−1^_ chow during the last 2 weeks. Mice in the groups Abx and Abx-Se received water containing a cocktail of broad-spectrum antibiotics (ampicillin 1g/L, neomycin 1g/L, metronidazole 1g/L, vancomycin 0.5 g/L) and the antifungal amphotericin B (10 mg/L) during the first week of the 3 weeks of treatment. Finally, mice were anesthetized by isoflurane inhalation and exsanguinated by cardiac puncture and dissected using a ceramic scalpel, and gut contents were collected from the large intestine (cecum and colon) and flash frozen in liquid nitrogen.

### 2.3 Untargeted Gut Metabolomics

#### 2.3.1 Sample Treatment

Gut samples were introduced into 1.5 mL plastic Eppendorf tubes for lyophilization and rehomogenized using a vortex. For the extraction of metabolites, 250 μL of methanol (MeOH) were added to 10 mg of gut sample and vortexed during 30 min. Mixtures were then centrifuged at 2057*g* and 4 °C for 10 min and the resulting supernatant was collected and taken to dryness using a speed vacuum system (Thermo Scientific Savant SPD111 V SpeedVac Concentrator) for 30 min at 45 °C. For GC-MS analysis, gut extracts were reconstituted with derivatizing reagents. For protection of carbonyl groups by a methoxymation reaction, dried extracts (2 mg, approximately) were redissolved in 50 μL of 20 mg mL^–1^ of methoxyamine in pyridine, and after briefly vortexing, the samples were incubated at 80 °C for 15 min. Subsequently, 50 μL of *N*-methyl-*N*-(trimethylsilyl)trifluoroacetamide (MSTFA) were added to the extract for silylation at 80 °C for 15 min. Finally, extracts were centrifuged at 2057*g* for 5 min. For UHPLC-QTOF-MS analysis, dried gut content extracts were redissolved in 50 μL of MeOH.

#### 2.3.2 GC-MS Analysis

Gas chromatographic analysis was performed in a Trace GC ULTRA gas chromatograph coupled to an ion trap mass spectrometer detector ITQ900 (Thermo Fisher Scientific), using a Factor Four capillary column VF-5MS 30 m × 0.25 mm ID, with 0.25 μm of film thickness (Agilent Technologies, Tokyo, Japan). The column temperature was set to 50 °C during 1 min and programmed to reach 310 °C at a rate of 10 °C per minute. This temperature was maintained during 10 min. A constant flow rate of 1 mL min^–1^ of helium as carrier gas was introduced into the system. Moreover, ionization was performed by electronic impact (EI) using 70 eV as voltage. The filament was off in the first 6 min of the chromatogram to avoid the signal of a large band from the solvent and other nonseparated peaks. Finally, full scan mode in the *m*/*z* range 35–650 was monitored and 1 μL of gut sample was injected in splitless mode.

#### 2.3.3 UHPLC-QTOF-MS Analysis

UHPLC-QTOF-MS analysis was carried out in an Agilent 1290 Series LC pump and Wellplate Autosampler coupled to an Agilent 6550 iFunnel Q-TOF LC/MS System equipped with a dual electrospray ion source operated in negative and positive mode (Agilent Technologies, Tokyo, Japan). An inverse phase chromatography with gradient was performed for the separation of metabolites. Water (A) and acetonitrile (B) with 0.1% formic acid were used as mobile phases. A flow rate of 0.4 mL min^–1^ running in a gradient method from 5 to 100% of phase B were selected for the analysis. Thus, 10 μL of extracted gut samples were injected to an Agilent Poroshell 120 EC-C18 column (100 × 3 mm; 1.8 μm; Agilent Technologies) thermostatted at 60 °C. For mass correction, the reference masses *m*/*z* 121.0509 and 922.0098, and *m*/*z* 112.9856 and 1033.9881 were constantly introduced into the system for positive and negative ionization modes, respectively. Full scan mode was monitored from 50 to 1100 *m*/*z*. The QTOF parameters were set to 3 kV for capillary voltage, 12 L min^–1^ at 250 °C for drying gas flow rate, and 52 psi for gas nebulizer. Fragmentor voltage was set to 175 V for positive and 250 V for negative ionization modes. A list containing the most significant features was imported and analyzed with the initial chromatographic conditions using the Agilent MassHunter Data Acquisition software in Targeted MS/MS mode with MS/MS scan rate of 1 spectrum s^–1^. Nitrogen was used as collision gas and several collision voltages were fixed from 10 to 40 V for the fragmentation of compounds. Data were acquired at centroid mode using a scan rate of 1.0 spectrum per second.

#### 2.3.4 Data Processing

GC-MS raw data processing was described previously.^26^ In brief, files were converted into CDF format using the Thermo File Converter tool (Thermo Fisher Scientific). XCMS software included in the R platform (http://www.r-project.org) was used for the extraction, alignment of peaks, and normalization. In this sense, data were extracted using the algorithm “matched filter method” which slices the data into extracted ion chromatograms (XIC) on a fixed step size, and then each slice was filtered with matched filtration using a second-derivative Gaussian as the model peak shape. The parameters for GC–MS data were S/N threshold 2, full width at half-maximum (fwhm) 3, and width of the *m*/*z* range 0.1. After peak extraction, grouping and retention time correction of peaks was realized in three iterative cycles with descending bandwidth (bw) from 5 to 1 s. For data normalization, the locally weighted scatter plot smoothing (LOESS) normalization method was performed, which adjusts the local median of log fold changes of peak intensities between samples in the data set to be approximately zero across the whole peak intensity range. The preprocessed data were then exported as a .csv file for further statistical analysis.

For UHPLC-QTOF-MS raw data processing was carried out with Agilent MassHunter Profinder B.10.0 software (Agilent Technologies). To extract the data, Batch Recursive Feature Extraction (RFE) for small molecules wizard from the software was applied. RFE performs two algorithms: First, the Molecular Feature Extraction algorithm (MFE) including extraction, selection of ion species, and charge state was used to find the features in the data set. Second, the initial features were aligned by retention time (RT) and mass, creating a list of unique features through binning. Then, the RT and mass data pairs of the aligned and binning features were used as input criteria to more accurately find the features using the Find by Ion algorithm (FbI). Additional filters such as scoring, integration, and peak filters were also applied to the data set. Table S1 shows the parameters and filters used for positive and negative modes. Moreover, Mass Profiler Professional B.10.0 (Agilent Technologies) was used for the normalization the data set using total area sums.

### 2.4 Statistical Analysis

GC-MS data were statistically processed with SIMCA-P software (version 11.5, published by UMetrics AB, Umeå, Sweden) and significant features were selected according to the Variable Importance in the Projection (VIP), considering only variables with VIP values higher than 1, indicative of significant differences among groups.

For UHPLC-QTOF-MS data processing Mass Profiler Professional B.10.0 (Agilent Technologies) was used for the determination of the most relevant metabolites between groups. For both features determined by GC-MS and UHPLC-QTOF-MS methodologies, principal component analysis (PCA) and partial least-squares discriminant analysis (PLS-DA) were carried out in order to compare the gut metabolomic profiles obtained. The predictive and class separation parameters *R*^2^ and *Q*^2^ of all models built were supplied by the software. Before performing statistical analysis, the data were submitted to Pareto scaling and logarithmic transformation.

One-way ANOVA and Tukey test for multiple comparisons were applied using STATISTICA 8.0 from StatSoft. Moreover, a Benjamini–Hochberg FDR correction was also applied to adjust the *p*-values. On the other hand, Spearman correlations between gut metabolites and microbiota at genus level and heatmaps were determined using R Software Package Hmisc (4.0.2 version).^31^ The level of statistical significance for all tests was set to *p* < 0.05.

#### 2.4.1 Annotation of Gut Metabolites

NIST Mass Spectral Library (version 08) was used to annotate the altered metabolites determined by GC-MS, considering a probability greater than 80%. Moreover, we selected a target ion and at least two identifier ions (qualifiers) from each metabolite mass spectrum. We chose ions with higher masses and intensities, because they are less affected by matrix. In addition, we checked the area qualifier/target ion ratio per metabolite and chose those with a variation less than 20%. We make sure that qualifier ions were specific for the metabolites. In addition, Kovats retention indexes (KRI) were calculated for altered metabolites using a mixture of alkanes (from C7 to C40, Sigma-Aldrich, Germany). For UHLC-QTOF-MS analysis, Agilent Qualitative Analysis Workflow MassHunter B.08.00 software was used to annotate the compounds. For this purpose, the workflow “Compound Discovery” and the compound mining “Find by Molecular Features” from the software was applied to the data set. METLIN (http://metlin.scripps.edu) and HMDB (http://hmdb.ca) databases were consulted for the annotation of altered compounds considering a score higher than 90%, which reflects how well the compound matches the mass, isotope pattern, and retention time of the target compound.

Moreover, MS-MS experiments were applied to samples in order to confirm the annotation of some compounds using a QTOF (6550 system, Agilent Technologies) with the same chromatographic conditions as applied for the primary analysis. Ions were targeted by collision-induced dissociation (CID) fragmentation on the fly based on the previously determined accurate mass and retention time.

### 2.5 Gut Microbiota Composition and Diversity

DNA extraction was carried out on approximately 100 g of gut content as described previously.^25^ Gut microbiota profile was determined by V3–V4 variable region of the 16S rRNA gene sequencing following Illumina protocols.^27^ Raw sequences were managed by use of in-house pipeline and by DADA2 pipeline.^28^ Taxonomic assignment was carried out by using Silva v132 database.^29^ Moreover, taxa present in a relative abundance less than 0.01% and those present less than 3 times in at least 20% of the samples were also filtered.

Data analysis, multivariate test, and data mining of microbiota were performed by Calypso web platform (v. 8.56).^30^ α- and β-diversity were obtained using this platform. Permutational multivariate analysis of variance (PERMANOVA) of Bray–Curtis distance was carried out. The visualization of the multivariate analysis was carried out by Redundancy Discriminant Analysis (RDA). Finally, data were classified by metadata factors and Wilcoxon test with False Discovery test Rate (FDR) for multiple test correction was used in order to evaluate differences in relative abundance.

## 3 Results

A combined analytical multiplatform based on GC-MS and UHPLC-QTOF-MS was applied to assess the impact of Se-supplementation on the gut metabolomes of C and Abx mice. In parallel, the gut microbiota was profiled to characterize the bidirectional interplay between them and altered metabolites.

### 3.1 Gut Metabolome Profiling by a GC-MS and UHPLC-QTOF-MS Platform

Different gut metabolome profiling was determined using the multiplatform based on GC-MS and UHPLC-QTOF-MS analysis, in positive and negative ionization modes (Figure S2). In order to ensure a good stability and reliable metabolomics results, the analysis was evaluated using a total of 6 quality control samples (QC), which consisted of a pull of all the gut samples included in the study. PCA plots showed a good clustering of the QCs samples (Figure S3). In addition, coefficient of variation (CV) of QCs was calculated for this purpose (Table S2), and only those compounds with a CV lower than 15% were considered in the study. Moreover, blanks were prepared using the same procedure as samples and analyzed at the beginning and at the end of the batch to evaluate the injector contamination and the presence of artifacts in GC-MS and UHPLC-QTOF-MS analysis (Figure S4).

**Figure 1:**
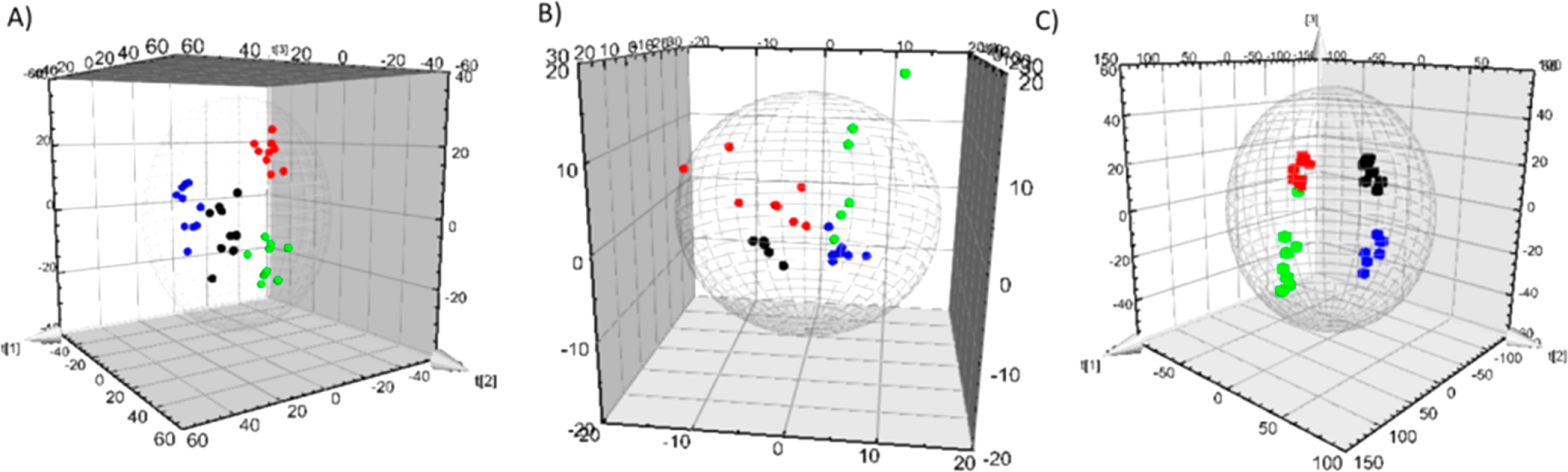
3D-PLS-DA of gut samples corresponding to (A) GC-MS analysis, (B) LC-ESI(+)-QTOF-MS, and (C) LC-ESI(−)-QTOF-MS. C: black dots, C-Se: red dots, Abx: blue dots, and Abx-Se: green dots.

PLS-DA showed good classifications between groups in both GC-MS and UHLC-QTOF-MS data set (Figure 1). Moreover, pairwise comparisons between C, C-Se, Abx, and Abx-Se were carried out to determine the metabolites responsible for the discrimination between groups. The 2D-PLS-DAs built from pairwise group comparison (Figure S5, S6, and S7) and values of *R*^2^ and *Q*^2^ from all the models built (Table S3) in the study were described in the Supporting Information

**Figure 2:**
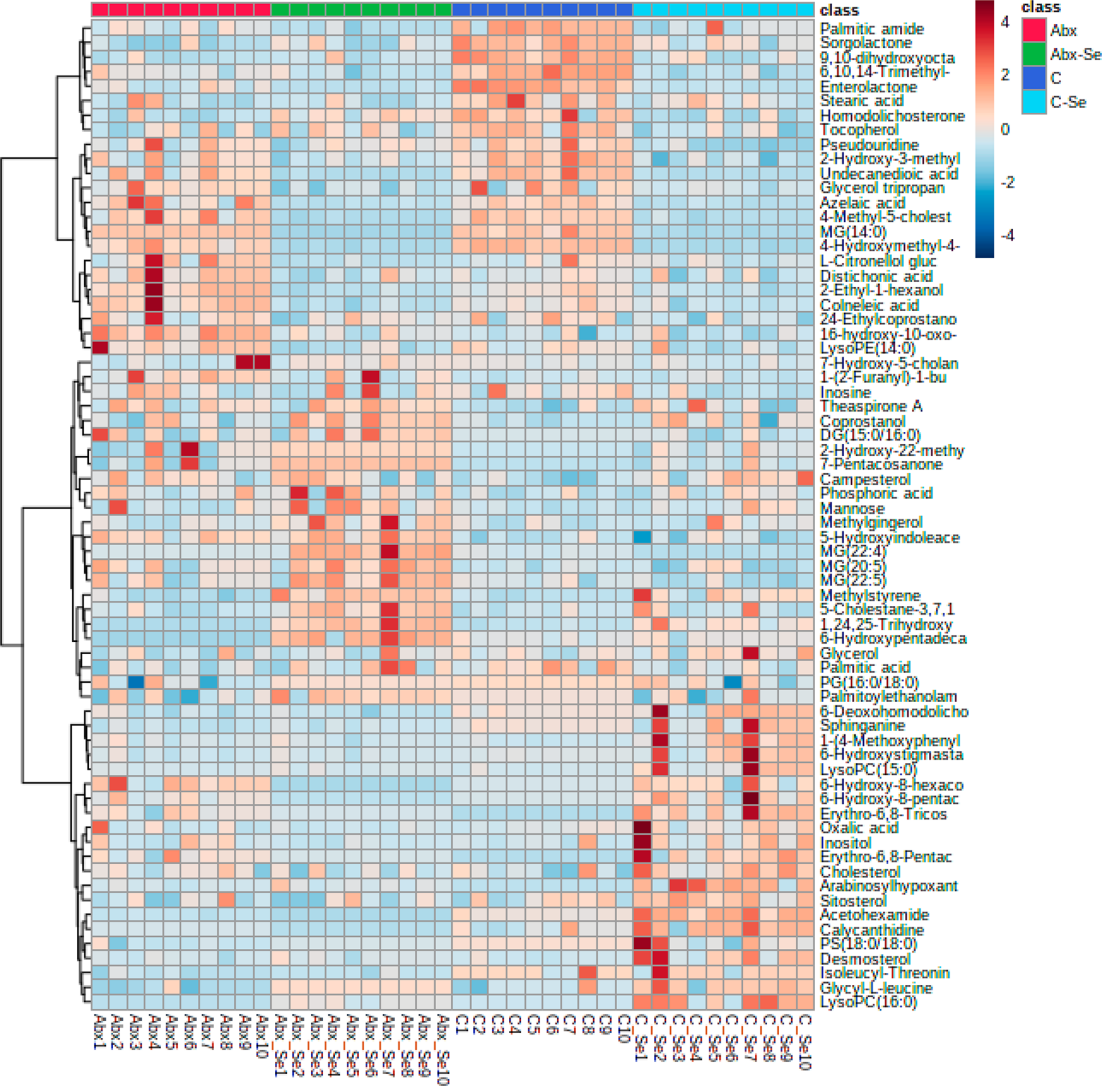
Cluster heatmap of gut metabolites from C, C-Se, Abx, and Abx-Se mice. Metabolites are represented in rows and mice groups in columns. Red and blue colors show increased and decreased levels of metabolites, respectively.

A total of 73 gut metabolites (Table S4) were annotated combining GC-MS (15 metabolites) and UHPLC-ESI(±)QTOF-MS (58 metabolites) analysis. KRIs of metabolites determined by GC-MS are summarized in Table S5. Figure 2 shows the abundance of the most significant metabolites in C, C-Se, Abx, and Abx-Se groups in a heatmap diagram.

The most altered classes of metabolites found in the groups of study were fatty acyls, glycerolipids, glycerophospholipids, prenol lipids, steroids, carboxylic acids, and organooxygen compounds (Table S4). As can be seen in Figure 2, there are two areas of metabolites that are different in conventional mice after Se-supplementation (C vs C-Se). Thus, we could observe an increased abundance (red color) of a group of metabolites including fatty acid alcohols, steroids, amino acids, and organooxygen compounds and decreased levels (blue color) of glycerolipids, fatty acids, fatty amides, and fatty alcohols in C-Se mice. Moreover, there is mainly one red area in Abx mice, which is blue in C, that represents metabolites increased by microbiota depletion. Interestingly, this area decreased after Se-supplementation (represented in blue color in Abx-Se). These metabolites are mainly related to fatty acids, prenol lipids, and glycerophospholipids compounds.

Specifically, 28 altered metabolites were found in C-Se against the control group, suggesting an influence of Se-supplementation diet on conventional mice metabolism. A decrease of fatty acids and conjugates such as stearic (0.49-fold), 9,10-dihydroxysteatic (0.27-fold), palmitic (0.69-fold), azelaic (0.20-fold), and undecaenoic acids (0.29-fold) was determined in gut samples of C-Se against C. Likewise, a significant decrease in the levels of monoglycerides (MGs), and a general increase of steroids, including sitosterol (1.61-fold), campesterol (2.44-fold), and coprostanol (1.16-fold) were found in the same group. We found the dysregulation of 32 metabolites in Abx group. In this sense, fatty acyls and prenol lipids were the most affected compounds. Levels of the fatty acids 16-hydroxy-10-oxohexadecanoic acid (1.94-fold), 6-hydroxy-8-pentacosanone (1.26-fold), and 2-hydroxy-22-methyltetracosanoic acid (1.61-fold) augmented in Abx. However, a decrease in stearic (0.39-fold) and palmitic acids (0.59-fold) were found in this group compared to C. On the other hand, the prenol lipids 6,10,14-trimethyl-5,9,13-pentadecatrien-2-one (0.61-fold), tocopherol (0.49-fold) and sorgolactone (0.24-fold) also decreased in Abx.

The highest number of altered metabolites (50 compounds) was found in Abx-Se compared to Abx. Levels of glycerolipids, glycerophospholipids, and steroids were significantly different after Se-supplementation in microbiota depleted mice, and some of their levels were close to those found in conventional mice. In this sense, a diminution in the glycerophospholipids lysophosphatidylethanolamine (14:0) (LPE, 0.28-fold), phosphatidylethanolamine (15:0/20:1) (PE, 0.43-fold), and phosphatidylglycerol (16:0/18:0) (PG, 0.36-fold) was observed in Abx-Se compared to Abx, while these compounds increased in Abx against C. Likewise, the steroids 7-hydroxy-5-cholanic acid (2.87-fold), 5-cholestane-3,7,12,23-tetrol (3.23-fold), coprostanol (1.24-fold), desmosterol (1.14-fold), campesterol (3.24-fold), 6-hydroxystigmasta-4,22-dien-3-one (1.10-fold), homodolichosterone (2.33-fold), and 1,24,25-trihydroxyvitamin D2 (2.16-fold) were found at higher levels in Abx-Se. Generally, these compounds decreased after depletion of microbiota in conventional mice. On the other hand, only 20 perturbed metabolites were found in Abx-Se against C. This fact could indicate that in spite of the depletion of microbiota by antibiotics, which led to the loss of a great number of metabolites, some of them can be recovered when aided by Se-supplementation. In this sense, perturbed levels of fatty acyls in Abx such as palmitic acid, 2-hydroxy-22-methyltetracosanoic acid, 6-hydroxy-8-pentacosanone, and the prenol lipids 6,10,14-trimethyl-5,9,13-pentadecatrien-2-one and tocopherol were not altered in Abx-Se group.

**Figure 3:**
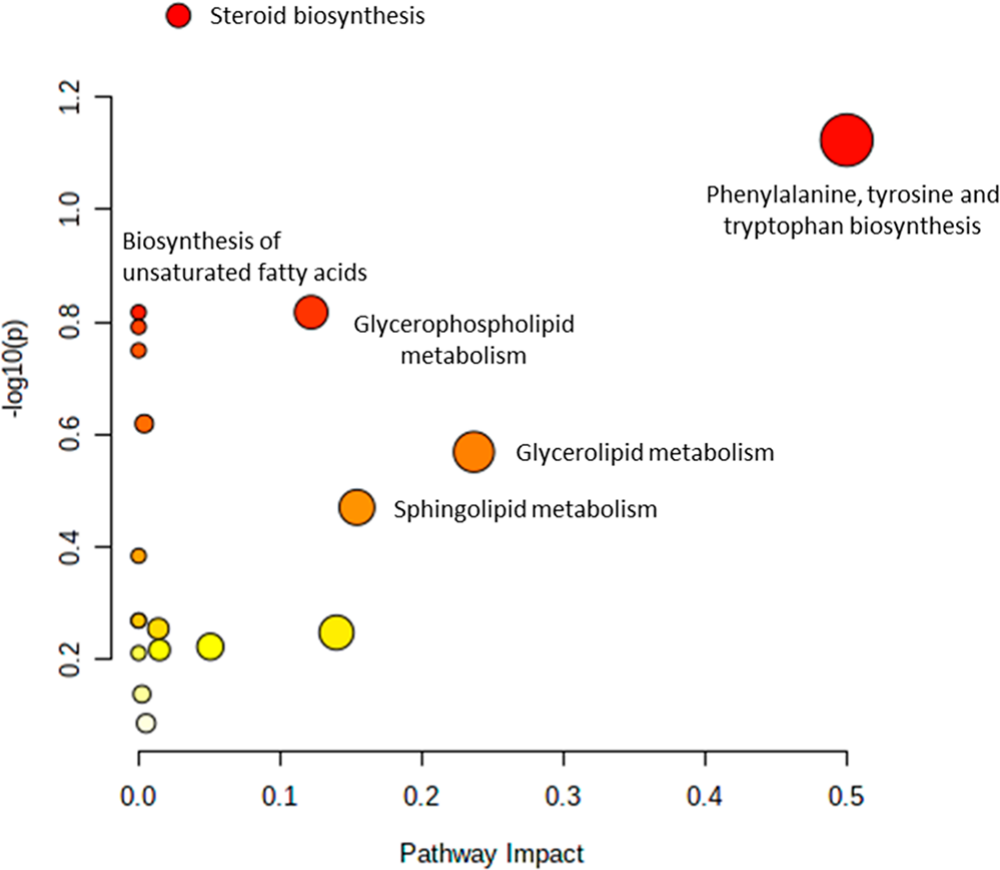
Pathway analysis plot indicating the most affected metabolic routes in Abx and Abx-Se groups. The *p*-value (indicated by a color gradient: from white (highest *p*-value) to red (lowest *p*-value)) is the *p* calculated from the enrichment analysis, and the impact (indicated by dot size) is the pathway impact value calculated from pathway topology analysis.

We also evaluated the most affected metabolic routes in Abx and Abx-Se groups using the available web tool MetaboAnalyst 5.0 (metaboanalyst.ca). The pathway analysis showed a total of 19 altered routes including steroid biosynthesis, phenylalanine, tyrosine, and tryptophan biosynthesis, biosynthesis of unsaturated fatty acids, glycerophospholipid metabolism, glycerolipid metabolism, and sphingolipid metabolism. Figure 3 shows the diagram of the pathway analysis, and Table S6 describes the numbers of hits, *p*-value, and the impact of the affected metabolic routes by the altered metabolites found in Abx and Abx-Se groups.

### 3.2 Gut Metabolite Profile Is Associated with Gut Microbiota Composition and Diversity

**Figure 4:**
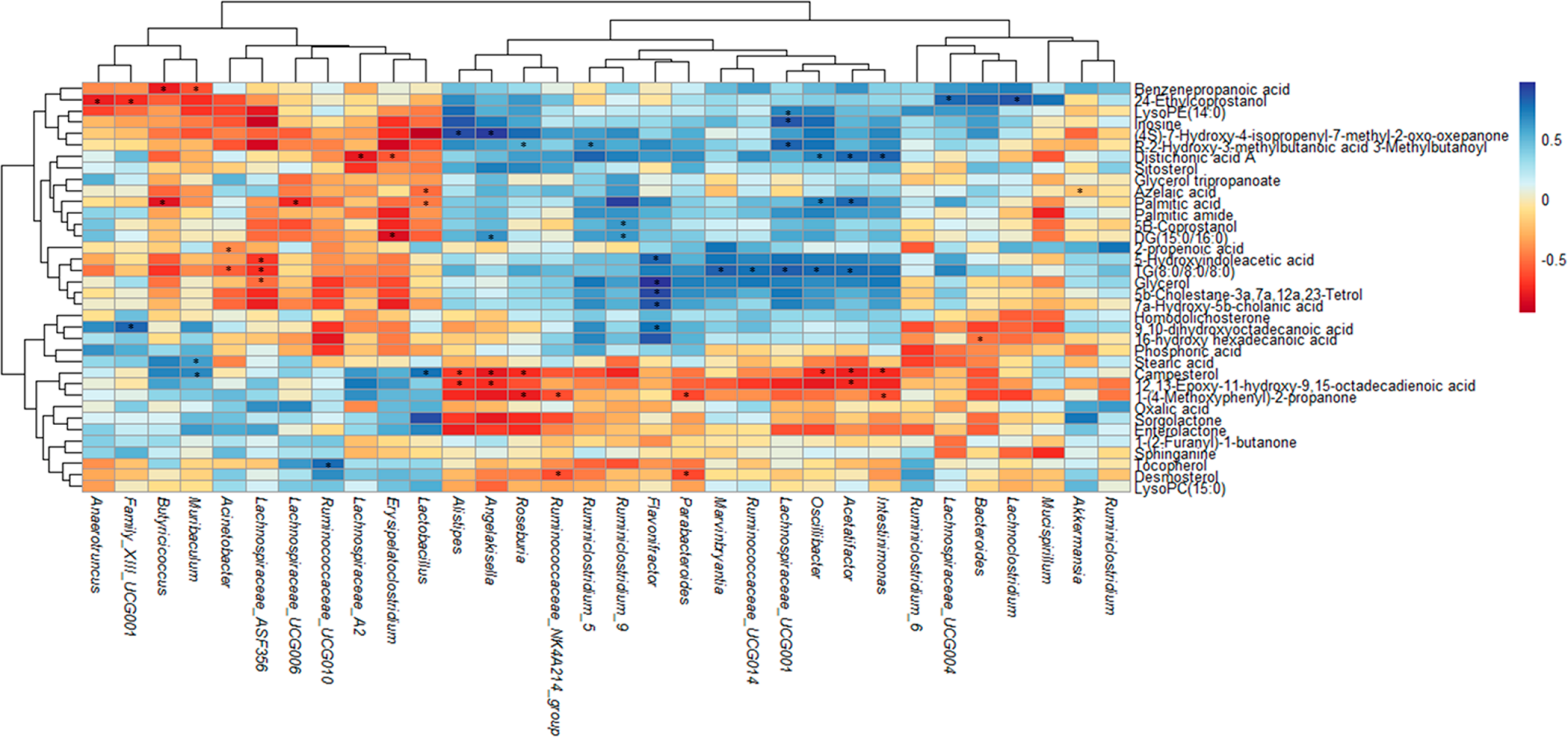
Spearman correlation heatmap analysis between altered gut metabolites and 32 top genuses (Abx-Se group).

In a previous work,^25^ we identified changes in the gut microbiota composition and diversity in mice after both Se-diet supplementation and/or antibiotic treatment. In addition, we reported the increase of the relative abundance of health-relevant taxa influenced by Se-intake.^25^ A total of 61 different genera and their relative abundance (Table S7) were determined in C, C-Se, Abx, and Abx-Se groups. Here, specific associations (Table S8) between gut metabolites and the relative abundance of identified bacterial genera in the different experimental groups were evaluated, our results suggesting that gut microbiota alterations are related to phenotype perturbations at the gut metabolome level. Thus, in the C group, we found negative correlations between different genera from *Lachnospiraceae* and *Ruminococcaceae* families (*Lachnospiraceae_FCS020_group, Lachnospiraceae_UCG001, Ruminiclostridium_9*, *Ruminococcaceae_UCG009*, and *Ruminococcaceae_UCG014*) and monoglycerolipids (MG(22:5), MG(20:5), MG(22:4)). Otherwise, we observed positive associations between monoglycerides and *Lachnospiraceae* members in C-Se (*Lachnospiraceae_UCG004* and *Lachnospiraceae_UCG006*). In Abx, prenol lipids (4-hydroxymethyl-4-methyl-5-cholesta-8,24-dien-3-ol and l-citronellol glucoside) were mainly positively correlated with *Family_XIII_UCG001*, *Flavonifractor*, *Oscillibacter*, *Ruminiclostridium*, and *Ruminiclostridium_9*, but the monoglycerolipids MG(22:5) and MG(20:5) and the purine nucleoside arabinosylhypoxanthine were negatively correlated with *Lachnospiraceae_NK4A136_group*, *Lachnospiraceae_UCG004*, *Ruminiclostridium_5, Ruminiclostridium_6*, *Ruminococcaceae_NK4A214_group*, and *Ruminococcaceae_UCG003* in the same group. Finally, positive associations between 1,24,25-trihydroxyvitamin D2, distichonic acid, pseudouridine, the monoglycerides MG(20,5), MG(14,0), and the taxa *Acetatifactor*, *Lachnospiraceae_UCG001, Flavonifractor*, *Intestinimonas*, *Marvinbryantia*, *Ruminiclostridium_5*, *Ruminococcaceae_NK4A214_group*, and *Ruminococcaceae_UCG014* were observed in Abx-Se (Figure 4). However, campesterol were mainly negatively correlated with *Acetatifactor*, *Alistipes*, *Angelakisella*, *Intestinimonas*, *Oscillibacter*, and *Ruminiclostridium_9* in this group.

## 4 Discussion

Although several studies have reported the effect of Se-intake on metabolism,^24,32,33^ even after exposure to xenobiotics,^2^ there are not many antecedents about the impact of Se supplementation diet on mice gut metabolism.^23^ In our work, we have studied for the first time the gut metabolomic profile of conventional and microbiota-depleted mice influenced by Se-supplementation including the associations between metabolic alterations and gut microbiota.

The most altered metabolomic profile was found in the Abx-Se group compared to Abx (50 altered metabolites of a total of 73 annotated). In contrast, very few differences were found between Abx-Se and C groups (Table S4).

Figure 2 shows one area of gut metabolites abundance remarkably increased in Abx vs C, which decreased in Abx-Se suggesting the potential beneficial role of Se-supplementation against antibiotic treatment consequences over these metabolites. Overall, levels of fatty acyls including fatty acids, fatty alcohols, and fatty acid esters increased in the Abx gut metabolome. This fact could be associated with an intestinal malabsorption maybe due to absence of microbes to transform these metabolites into energy supply.^34^ The levels of these fatty acids decreased after Se-supplementation in Abx pretreated mice, suggesting a beneficial effect of Se on the intestinal microbiota restoration that resumes the absorption of these compounds. Concretely, the levels of 16-hydroxy-10-oxohexadecanoic acid, 6-hydroxy-8-pentacosanone, 6-hydroxy-8-hexacosanone, 2-hydroxy-22-methyltetracosanoic acid, erythro-6,8-tricosanediol, and erythro-6,8-pentacosanediol were significantly altered in Abx vs C, but no significant changes were observed in Abx-Se vs C.

According to these results, the pathway analysis showed that the unsaturated fatty acid biosynthesis was one of the most affected metabolic routes by the exposure of antibiotics and Se-supplementation (Figure 3). In addition, fatty acids and their metabolites play important roles in the intestinal immune system^35^ and are crucial for the health of the host.^36^ Moreover, increased levels of azelaic acid are linked to oxidative stress and depression.^16^ Higher levels of azelaic acid were found in Abx mice, and these levels diminished after Se-intake in Abx-Se suggesting that dietary Se-supplementation has a beneficial role against oxidative stress on metabolism, which is one of the most known properties of Se. The majority of the studies about the gut microbiota and their impact in fatty acids metabolism are related to the production of short-chain fatty acids (SCFAs), such as acetate, propionate and butyrate by carbohydrate fermentation. It is well-known that SCFAs are essential for providing energy to epithelial cells^37^ and have beneficial effects in the maintenance of the gut barrier.^38^ However, we did not find alteration in the levels of SCFAs in our study. These metabolites probably could not be detected because they eluted during the time the filament was turned off (first 6 min) in the chromatograph. Otherwise, few works have described the role of gut microbiota in the metabolism of long chain fatty acids (LCFAs), but some of them have reported the effect of LCFAs intake, including palmitic acid, on gut microbiota composition.^39^ In our work, levels of the LCFAs 6-hydroxypentadecanedioic acid, 12,13-epoxy-11-hydroxy-9,15-octadecadienoic acid, 9,10-dihydroxystearic acid, palmitic acid, and stearic acid diminished in Abx and Abx-Se groups against C, increasing in Abx-Se against Abx, suggesting an influence of Se-supplementation in LCFA metabolism on microbiota depleted mice. Steroid biosynthesis was the main affected metabolic route due to the depletion of microbiota, and also to Se-supplementation (Figure 3). Most of the steroids such as cholesterol, 4-methyl-5-cholestan-8,24-dien-3-ona, coprostanol, 24-ethylcoprostanol, demosterol, and campesterol were found at significantly higher levels in Abx group. It is well-known that nonabsorbed sterols reach the colon, where gut microbiota biotransform them in subproducts.^40^ Sterol biotransformation by the gut microbiota is important for the production of metabolites involved in the energy supply to the host through anaerobic fermentative processes.^40^ Low levels of the prenol lipids such as tocopherol, 6,10,14-trimethyl-5,9,13-pentadecatrien-2-one, 4-hydroxymethyl-4-methyl-5-cholestan-8,24-dien-3-ol, and sorgolactone were observed in Abx mice. The antioxidant activity of prenol lipids have been reported in the literature.^41^ In our study, prenol lipids also decreased after microbiota depletion (Abx vs C) and increased after Se-supplementation (Abx-Se) indicating that the well-known role of Se against oxidative stress^1^ could be mediated, almost in part, by gut microbiota. Differences in indole derivatives compounds were observed in Abx and Abx-Se groups. In this sense, we found significant increased levels of 5-hydroxyindoleacetic in Abx-Se, and decreased levels of calycanthidine in Abx. It is known that indole derivatives are crucial in the interplay between host and microbiota, which are involved in tryptophan metabolism and, consequently, related to the microbiota-gut-brain axis.^42^ The pathway analysis (Figure 3) also showed perturbations in phenylalanine, tyrosine, and tryptophan metabolism. Changes in the levels of these amino acids by gut microbiota, especially tryptophan, have been related to alterations in the functioning of central and enteric nervous systems.^43^

In the same way, significantly augmented and decreased levels of the bile acid 7-hydroxy-5-cholanic acid were found in Abx-Se and Abx groups, respectively. As indole derivatives, many authors have reported the role of bile acids in the link between the intestinal microbiome and the brain. These compounds have been recognized as signaling molecules for a variety of metabolic activities.^44,45^ According to the pathway analysis (Figure 3), glycerophospholipid and glycerolipids metabolisms were significantly affected in Abx and Abx-Se mice. It is known that gut microbiota has been shown to affect lipid metabolism.^46^ In our work, significant differences between levels of glycerides (MG(14:0), MG(20:5), MG(22:5), MG(22:4), and DG(15:0/16:0)) and phospholipids (LPC(16:0), LPE(14:0) PE(15:0/20:1), PG(16:0/18:0), and PS(18:0/18:0)) were found in Abx-Se group against Abx. Specifically, the levels of MG(22:4), LPC(15:0), PG(16,0/18:0), and PS(18,0/18:0) were found altered in Abx vs C; however, no significant changes in the levels of these compounds were observed between Abx-Se and C, suggesting a potential effect of Se in the microbiota restoration, which positively affects the glycerophospholipids and glycerolipids metabolisms. In the same way, the organooxygen compounds 1-(2-furanyl)-1-butanone, theaspirone A, and 7-pentacosanone were found significantly perturbed in Abx mice, but no alteration were observed in Abx-Se compared to C.

As previously commented, we reported differences in gut microbiota composition depending on Se-supplementation and antibiotic exposure.^25^ We also concluded that Se can increase the relative abundance of some health-relevant taxa such as *Christensenellaceae*, *Ruminococcaceae*, and *Lactobacillus.*(25) The genus *Lactobacillus*, which includes many bacterial strains considered as potential probiotics,^47^ was positively correlated with the metabolites l-citronellol glucoside and PG(16:0/18:0) in the C-Se group. In the same way, *Lactobacillus* was positively correlated with 6,10,14-trimethyl-5,9,13-pentadecatrien-2-one and campesterol, and negatively with fatty acids (azelaic acid and stearic acid) in Abx-Se mice. No correlations between *Lactobacillus* and gut metabolites were observed in Abx.^48^ On the other hand, although some *Lachnospiraceae* groups were mainly negatively associated with monoglycerides in C group, we observed positive correlations between the genera *Lachnospiraceae_UCG004* and *Lachnospiraceae_UCG006* and the monoglycerides MG(20:5), MG(22:4), MG(22:5) after Se-supplementation (C-Se), suggesting the impact of Se-intake on glycerolipid metabolism through the function of *Lachnospiraceae* members. It is known that *Lachnospiraceae* members are involved in the production of butyrate^14^ and consequently in the SCFA metabolism. Moreover, some authors have described that *Lachnospiraceae* are related to changes in lipid metabolism, as well as specific nutrients.^49^ Likewise, we found that *Flavonifactor* and *Oscillibacter* members from the *Ruminococcaceae* family, which was influenced by Se-supplementation, were positively correlated with steroids (1,24,25-trihydroxyvitamin D2, 5-cholestane-3,7,12,23-tetrol, and 4-hydroxymethyl-4-methyl-5-cholesta-8,24-dien-3-oland) in Abx-Se mice. Some authors have reported the link between the stimulation of *Ruminococcaceae* species and Se-yeast supplementation, contributing to the maintenance of gut health and providing enzymatic ability to degrade cellulose and hemicellulose.^23,48^

## 5 Conclusions

The combined analytical multiplatform based on GC-MS and UHPLC-QTOF-MS is a powerful tool that has allowed us to determine a wide variety of intestinal metabolites. A total of 73 metabolites were annotated in gut content from the different mice groups. We observed that most of the gut metabolites annotated (70%) were altered after Se-supplementation of microbiota-depleted mice (Abx-Se vs Abx) and only few metabolites (30%) showed differences between this group and conventional mice (Abx-Se vs C). This fact may indicate that Se-supplementation considerably affects gut metabolome, as well as gut microbiota profile, even after microbiota depletion (e.g., ↑ *Lactobacillus* genus, ↓ fatty acids related to malabsorption, ↓ metabolites related to oxidative stress). Novel and important associations have been determined for the first time between gut metabolites and gut microbiota in mice fed an Se-supplemented diet. A wide panel of altered metabolites was detected in each group, including also gut–brain axis metabolites, which provide valuable information about the mechanisms by which Se-supplementation affects metabolism.

## Supporting Information Available

Figure S1: Experimental design of the study; Figure S2: Metabolomic profiles of gut samples from GC-MS analysis, UHLC-ESI(+)-QTOF-MS, and UHPLC-ESI(−)-QTOF-MS; Figure S3: 3D-PCA of gut samples determined by GC-MS, UHPLC-ESI(+)-QTOF-MS, and UHPLC-ESI(−)-QTOF-MS; Figure S4: Blank samples from GC-MS analysis, UHLC-ESI(+)-QTOF-MS, and UHPLC-ESI(−)-QTOF-MS; Figure S5: 2D-PLS-DA of pairwise comparisons of gut samples from C, C-Se, Abx, and Abx-Se, groups determined by GC-MS; Figure S6: 2D-PLS-DA of pairwise comparisons of gut samples from C, C-Se, Abx, and Abx-Se determined by ESI-(+)-UHPLC-MS; Figure S7: 2D-PLS-DA of pairwise comparisons of gut samples from C, C-Se, Abx, and Abx-Se groups determined by ESI-(−)-UHPLC-MS; Table S1: Batch Recursive Feature Extraction parameters (UHPLC-QTOF-MS analysis); Table S2: Coefficient of variation (CV) of gut metabolites calculated in quality control (QC) samples; Table S3: Q2 and R2 values from PLS-DA of C, C-Se, Abx, and Abx-Se groups; Table S4: Gut altered metabolites ordered by class; Table S5: Kovats retention Index (KRI) of GC-MS metabolites; Table S6: Pathway analysis details of altered metabolites in Abx and Abx-Se groups; Table S7: Taxa Abundance at genus level in C, C-Se, Abx, and Abx-Se groups; Table S8: Spearman correlation coefficient (rho) of altered gut metabolites and genus (PDF)

## Abbreviations

C: control mice group fed rodent diet
C-Se: mice group fed Se-supplementation diet
Abx: antibiotic treated mice fed rodent diet
Abx-Se: antibiotic treated mice fed Se-supplementation diet
GC: gas chromatography
UHPLC: ultrahigh performance liquid chromatography
MS: mass spectrometry
QTOF: quadrupole time-of-flight analyzer
ESI: electrospray ionization.
