# Supplementary material for: Untargeted Gut Metabolomics to Delve the Interplay between Selenium Supplementation and Gut Microbiota
Source: J Proteome Res. 2021 Nov 4;21(3):758–67. doi: 10.1021/acs.jproteome.1c00411 (PMC8902802; doi:10.1021/acs.jproteome.1c00411)
Supplement: Supplementary file 1 — pr1c00411_si_001.pdf [file pr1c00411_si_001.pdf]

# Supporting Information

## Untargeted gut metabolomics to delve the interplay between selenium supplementation and gut microbiota

Belén. Callejón-Leblic[1], Marta Selma-Royo[2], María Carmen Collado[2], José Luis Gómez-Ariza[1], Nieves Abril[3], Tamara García-Barrera*[1]

1. Research Center of Natural Resources, Health and the Environment (RENSMA). Department of Chemistry, Faculty of Experimental Sciences, Campus El Carmen, University of Huelva, Fuerzas Armadas Ave., 21007, Huelva, Spain.

2. Institute of Agrochemistry and Food Technology-National Research Council (IATA-CSIC), Department of Biotechnology, Agustin Escardino 7. 46980 Paterna, Valencia, Spain.

3. Department of Biochemistry and Molecular Biology, University of Córdoba, Campus de Rabanales, Edificio Severo Ochoa, E-14071, Córdoba, Spain.

*** Corresponding Author**

E-mail: tamara@dqcm.uhu.es

| Supporting Components | |
|---|---|
| Figure S1 | Experimental design of the study |
| Figure S2 | Metabolomic profiles of gut samples from (a) GC-MS analysis, (b) UHLC-ESI(+)-QTOF-MS and (c) UHPLC-ESI(-)-QTOF-MS. |
| Figure S3 | 3D-PCA of gut samples determined by (a) GC-MS, (b) UHPLC-ESI(+)-QTOF-MS and (c) UHPLC-ESI(-)-QTOF-MS. |
| Figure S4 | Blank samples from (a) GC-MS analysis, (b) UHLC-ESI(+)-QTOF-MS and (c) UHPLC-ESI(-)-QTOF-MS. |
| Figure S5 | 2D-PLS-DA of pairwise comparisons of gut samples from C (black dots), C-Se (red dots), Abx (blue dots), and Abx-Se (green dots), groups determined by GC-MS. (a) PLS-DA of C versus C-Se; (b) PLS-DA of C versus Abx; (c) PLS-DA of C versus Abx-Se; (d) PLS-DA of Abx versus Abx-Se. |
| Figure S6 | 2D-PLS-DA of pairwise comparisons of gut samples from C (black dots), C-Se (red dots), Abx (blue dots), and Abx-Se (green dots) determined by ESI-(+)-UHPLC-MS. (a) PLS-DA of C versus C-Se; (b) PLS-DA of C versus Abx; (c) PLS-DA of C versus Abx-Se; d) PLS-DA of Abx versus Abx-Se |
| Figure S7 | 2D-PLS-DA of pairwise comparisons of gut samples from C, C-Se, Abx and Abx-Se groups determined by ESI-(-)-UHPLC-MS. (a) PLS-DA of C versus C-Se; (b) PLS-DA of C versus Abx; (c) PLS-DA of C versus Abx-Se; (d) PLS-DA of Abx versus Abx-Se. |
| Table S1 | Batch Recursive Feature Extraction parameters (UHPLC-QTOF-MS analysis). |
| Table S2 | Coefficient of variation (CV) of gut metabolites calculated in quality control (QC) samples. |
| Table S3 | $Q^2$ and $R^2$ values from PLS-DA of C, C-Se, Abx and Abx-Se groups. |
| Table S4 | Gut altered metabolites. |
| Table S5 | Kovat´s retention Index (KRI) of GC-MS metabolites. |
| Table S6 | Pathway analysis details of altered metabolites in Abx and Abx-Se groups. |
| Table S7 | Taxa Abundance at genus level in C, C-Se, Abx and Abx-Se groups |
| Table S8 | Spearman correlation coefficient (rho) of altered gut metabolites and genus. |

Figure S1. Experimental design of the study

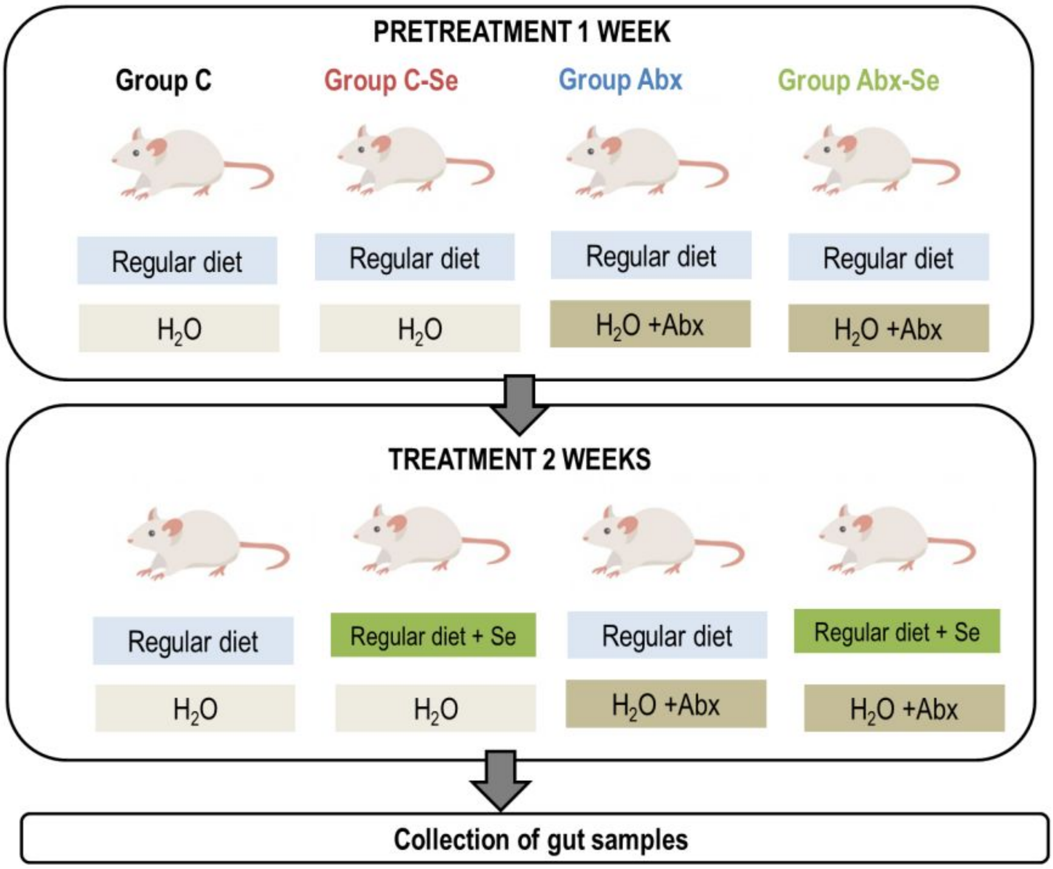

Figure S2. Metabolomic profiles of gut samples from (a) GC-MS analysis, (b) UHLC-ESI(+)-QTOF-MS and (c) UHPLC-ESI(-)-QTOF-MS.

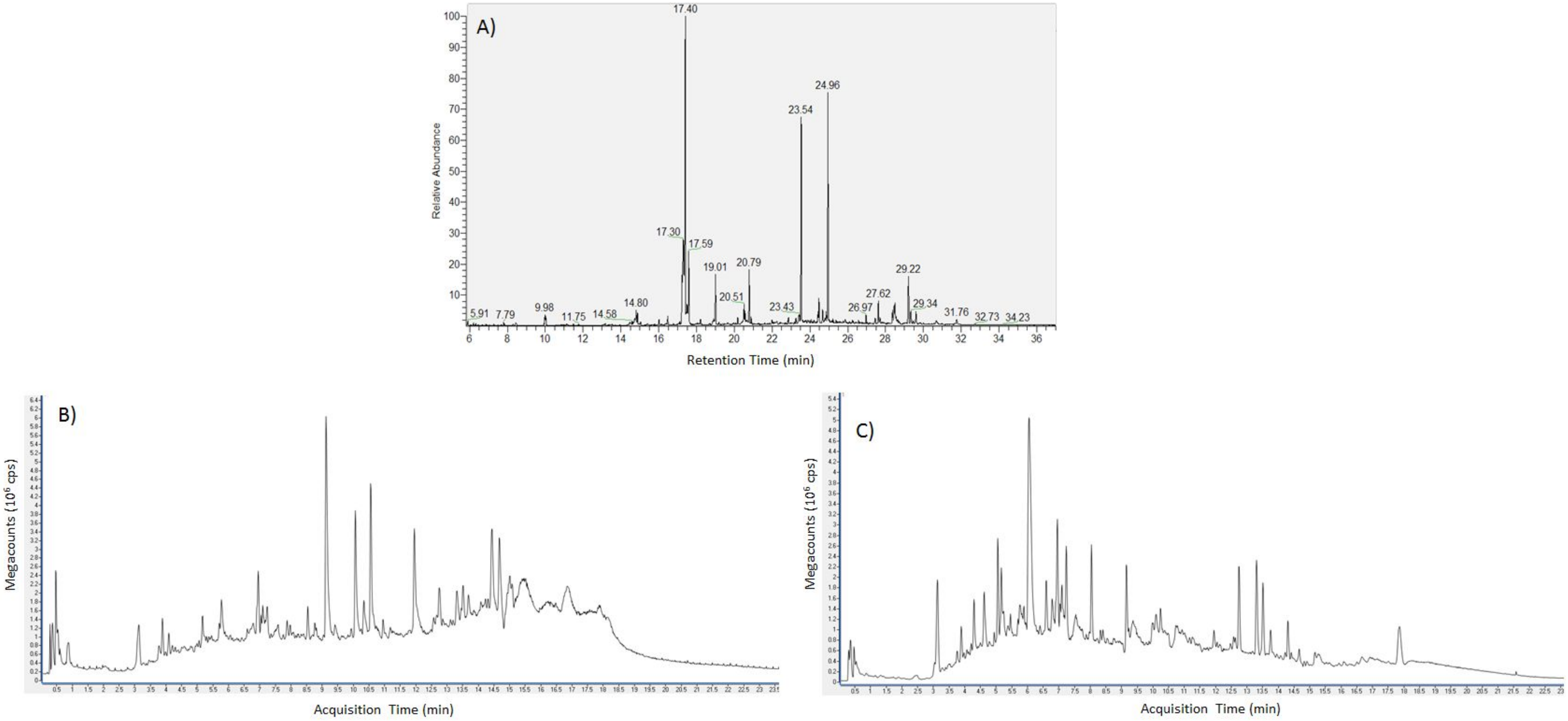

Figure S3. 3D-PCA of gut samples determined by (a) GC-MS, (b) UHPLC-ESI(+)-QTOF-MS and (c) UHPLC-ESI(-)-QTOF-MS. C (black triangles), C-Se (red triangles), Abx (blue triangles), Abx-Se (green triangles) and QCs ( yellow triangles ).

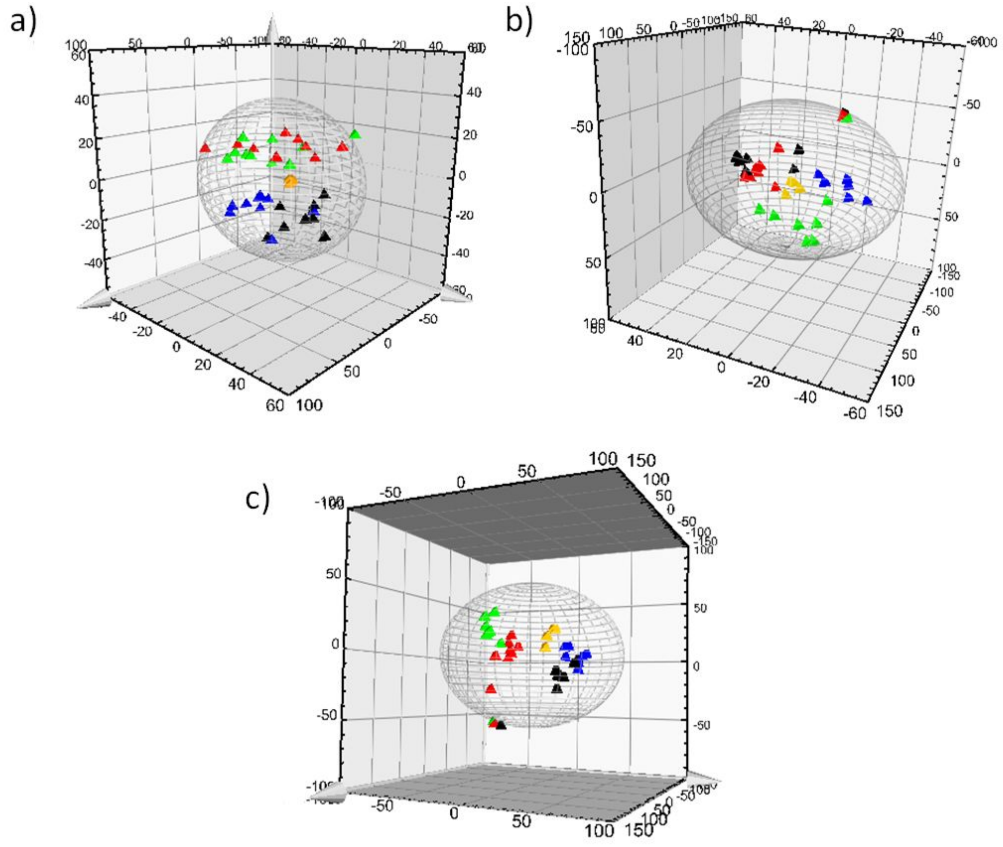

Figure S4. Blank samples from (a) GC-MS analysis, (b) UHLC-ESI(+)-QTOF-MS and (c) UHPLC-ESI(-)-QTOF-MS.

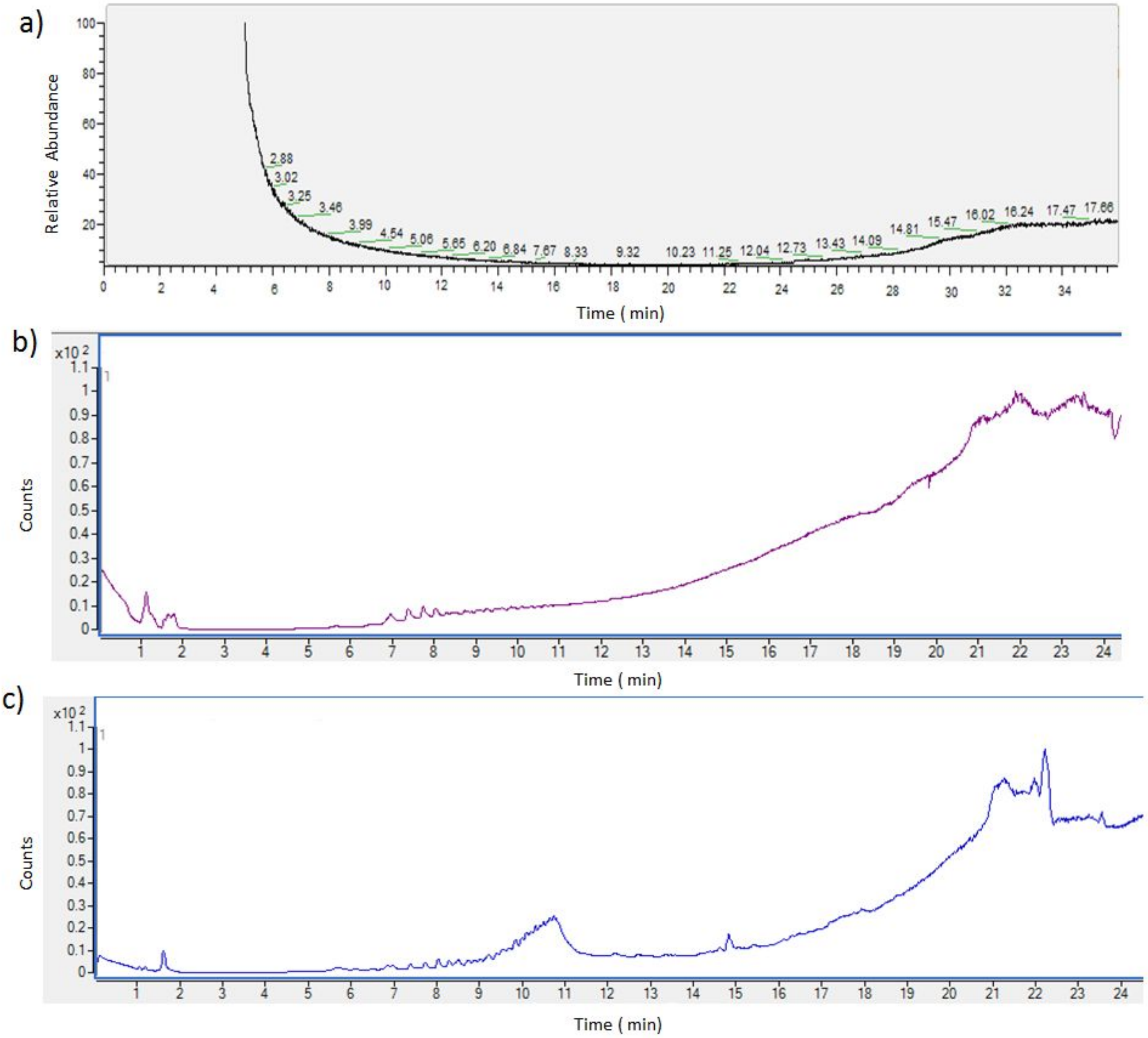

Figure S5. 2D-PLS-DA of pairwise comparisons of gut samples from C (black dots), C-Se (red dots), Abx (blue dots), and Abx-Se (green dots), groups determined by GC-MS. (a) PLS-DA of C versus C-Se; (b) PLS-DA of C versus Abx; (c) PLS-DA of C versus Abx-Se; (d) PLS-DA of Abx versus Abx-Se.

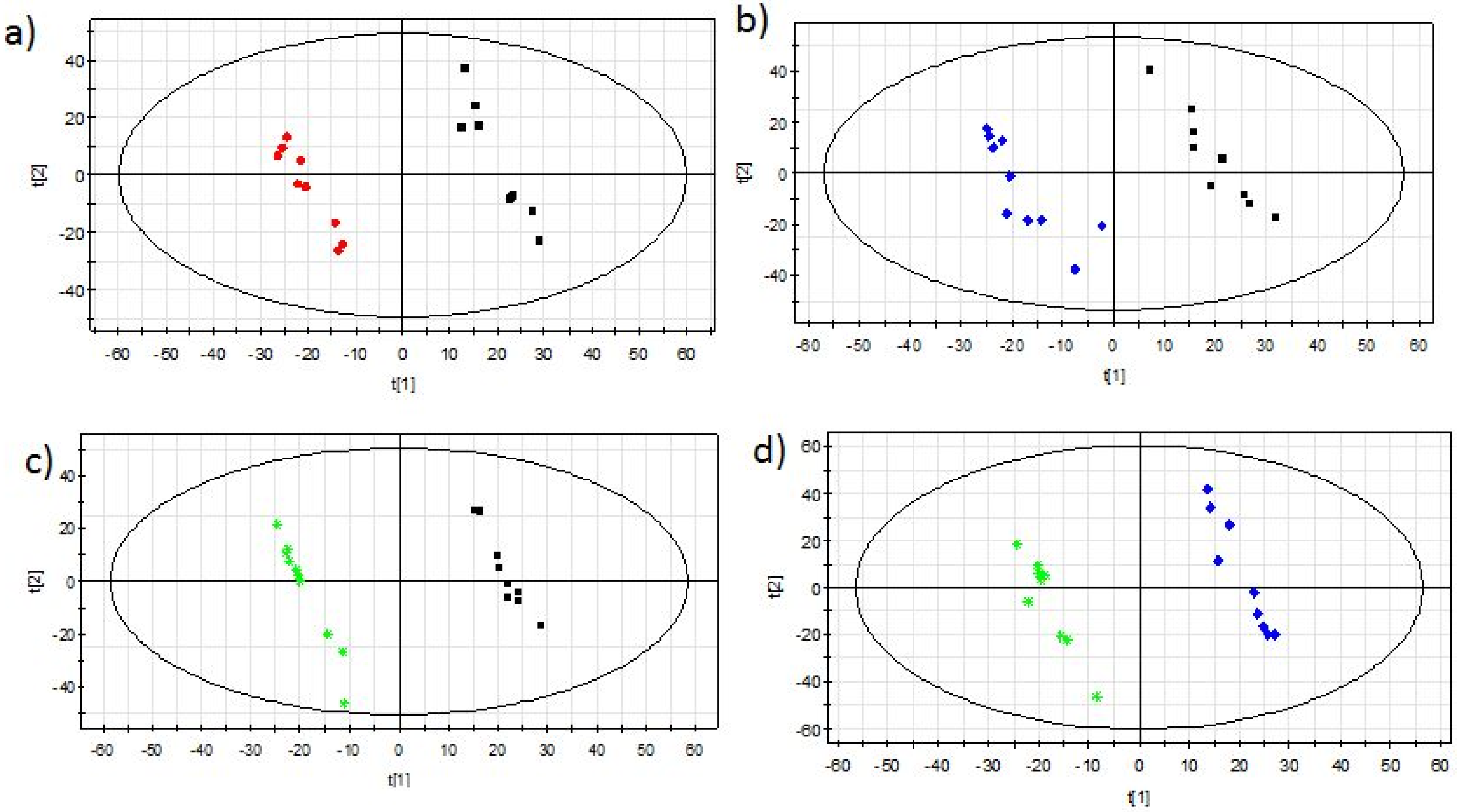

Figure S6. 2D-PLS-DA of  pairwise comparisons of gut samples from C (black dots), C-Se (red dots), Abx (blue dots), and Abx-Se (green dots) determined by ESI-(+)-UHPLC-MS. (a) PLS-DA of C versus C-Se; (b) PLS-DA of C versus Abx; (c) PLS-DA of C versus Abx-Se; d) PLS-DA of Abx versus Abx-Se.

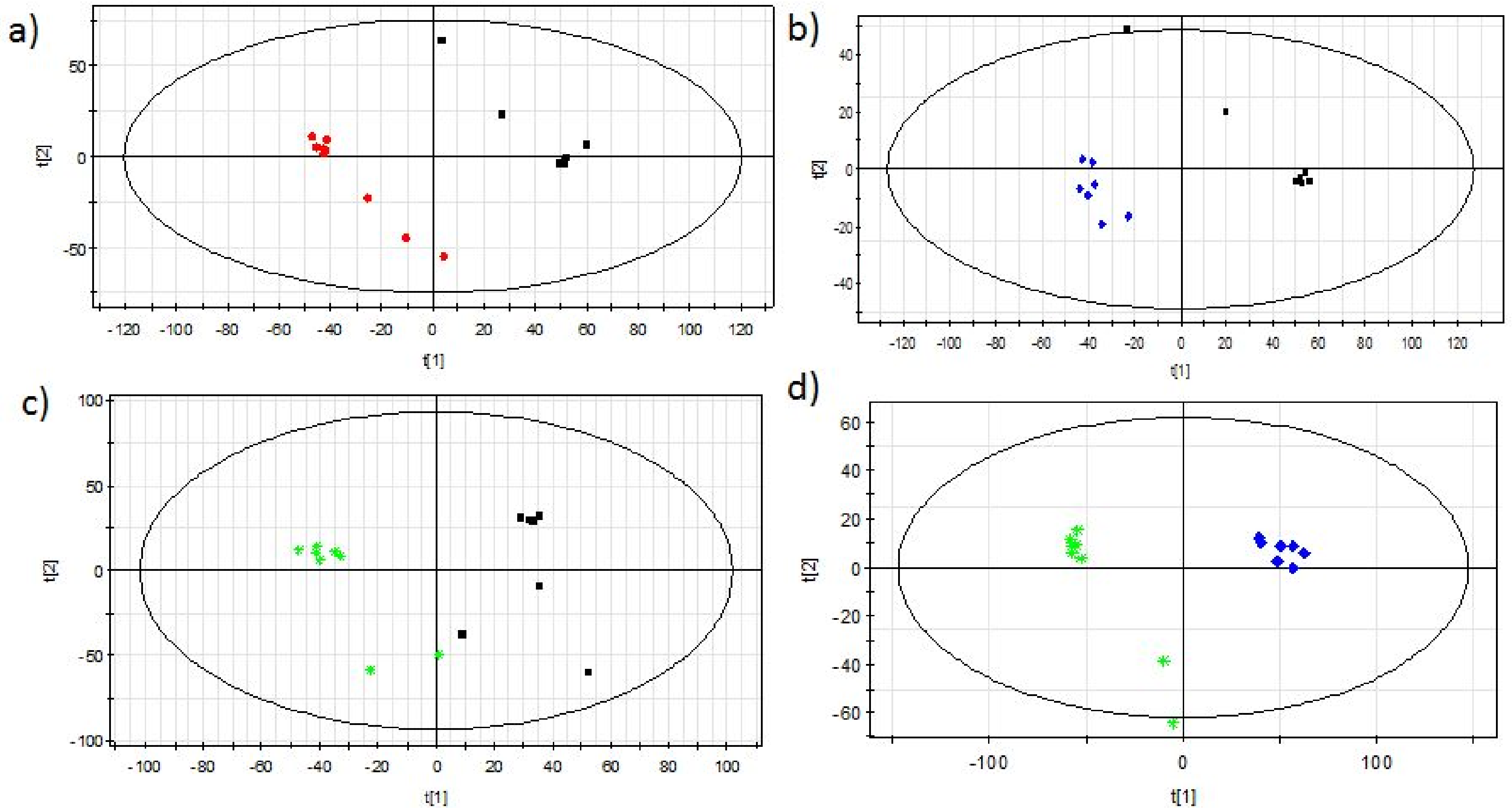

Figure S7. 2D-PLS-DA of pairwise comparisons of gut samples from C, C-Se, Abx and Abx-Se groups determined by ESI-(-)-UHPLC-MS. A) PLS-DA of C versus C-Se groups; b) PLS-DA of C versus Abx groups; c) PLS-DA of C versus Abx-Se groups; d) PLS-DA of Abx versus Abx-Se groups

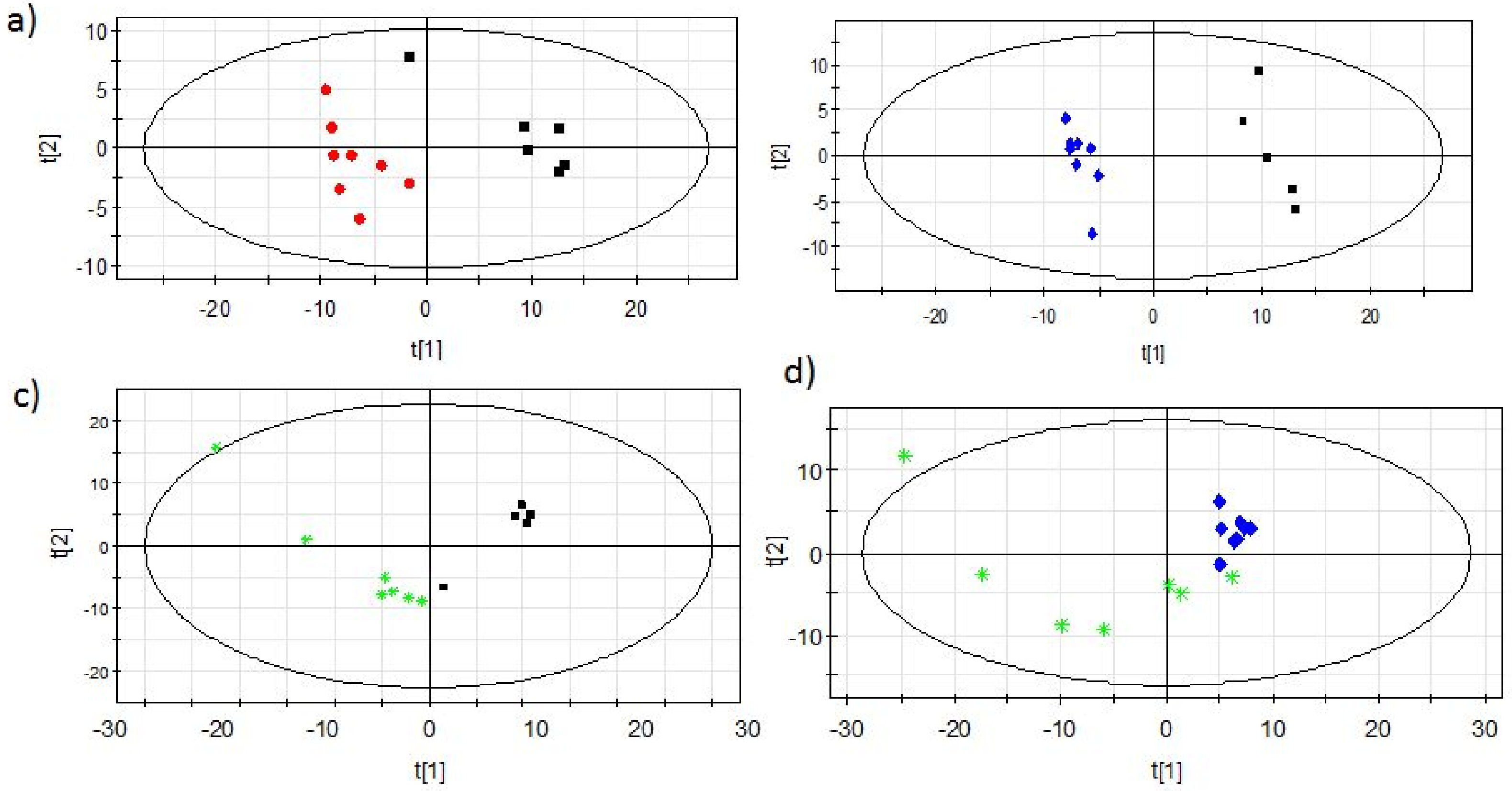

Table S1. Batch Recursive Feature Extraction parameters (UHPLC-QTOF-MS analysis)

| **Batch Recursive Feature Extraction** | |
|---|---|
| **Extraction** | |
| Peak weight | > 600 counts |
| **Ion species** | |
| Positive Mode | $[M+H]^+$, $[M+Na]^+$ |
| Negative Mode | $[M-H]^-$, $[M-Cl]^-$ |
| **Integration** | Agile 2 |
| **Alignment Parameters** | |
| RT Tolerance | 0.00% ± 0.3 min |
| Mass Tolerance | 20 ppm ± 2.00 mDa |
| **Molecular Feature Extraction Filters** | |
| Score (MFE) | > 90 |
| **Tolerance and EIC** | |
| Masses | ± 10.00 ppm |
| RT | ± 0.300 min |
| Possible m/z | Symmetric (ppm) |
| **Peak Filter** | |
| Absolute area | > 20000 counts |
| **Chromatogram Format** | **Centroid** |
| **Peak Spectrum** | |
| Average scans | 10 % of peak height |
| **Find by ion filters** | |
| Score (Tgt) | > 90 |
| **Entities** | |
| Number of entities found | Positive Mode: 333 |
| | Negative Mode: 1218 |
| Number of significant entities found | Positive Mode: 333 |
| | Negative Mode: 155 |

Table S2. Coefficient of variation (CV) of gut metabolites calculated in quality control (QC) samples. Lysophosphatidylcholine (LPC), lysophosphatidylethanolamine (LPE), phosphatidylethanolamine (PE); phosphatidylglycerol (PG); phosphatidylserine (PS); monoglyceride (MG)

| Compound | % CV in QC |
|---|---|
| 6-Hydroxystigmasta-4,22-dien-3-one | 1.5 |
| 9,10-Dihydroxystearic acid | 8.7 |
| 1-(2-Furanyl)-1-butanone | 0.7 |
| 1-(4-Methoxyphenyl)-2-propanone | 2.9 |
| 12,13-Epoxy-11-hydroxy-9,15-octadecadienoic acid | 4.0 |
| 16-Hydroxy-10-oxohexadecanoic acid | 0.6 |
| 1,24,25-Trihydroxyvitamin D2 | 1.2 |
| 24-Ethylcoprostanol | 12.4 |
| 2-Ethyl-1-hexanol sulfate | 1.1 |
| 2-Hydroxy-22-methyltetracosanoic acid | 0.5 |
| 3-Hydroxyisoheptanoic acid | 2.5 |
| 4-Hydroxymethyl-4-methyl-5-cholesta-8,24-dien-3-ol | 2.0 |
| 4-Methyl-5-cholesta-8,24-dien-3-one | 2.4 |
| 5-Cholestane-3,7,12,23-Tetrol | 1.8 |
| Coprostanol | 3.8 |
| 5-Hydroxyindoleacetic acid | 0.5 |
| 6,10,14-Trimethyl-5,9,13-pentadecatrien-2-one | 3.3 |
| 6-Deoxohomodolichosterone | 3.2 |
| 6-Hydroxy-8-hexacosanone | 0.6 |
| 6-Hydroxy-8-pentacosanone | 0.3 |
| 6-Hydroxypentadecanedioic acid | 0.3 |
| 7-Hydroxy-5-cholanic acid | 8.3 |
| 7-Pentacosanone | 0.4 |
| Acetohexamide | 0.5 |
| Methylstyrene | 3.3 |
| Arabinosylhypoxanthine | 0.1 |
| Ascorbyl stearate | 0.9 |
| Azelaic acid | 13.1 |
| Calycanthidine | 0.6 |
| Campesterol | 13.2 |
| Cholesterol | 6.7 |
| Colneleic acid | 4.6 |
| Desmosterol | 2.7 |
| DG(15:0/16:0) | 1.2 |
| Distichonic acid | 0.7 |
| Enterolactone | 4.6 |
| Erythro-6,8-Pentacosanediol | 0.1 |

| | |
|---|---|
| Erythro-6,8-Tricosanediol | 0.4 |
| Ethyl 3-oxobutanoate | 0.7 |
| Glycerol | 11.7 |
| Glycerol tripropanoate | 2.6 |
| Glycyl-L-leucine | 1.0 |
| Homodolichosterone | 12.4 |
| Inosine | 10.7 |
| Isoleucyl-Threonine | 0.1 |
| L-Citronellol glucoside | 6.4 |
| LPC(15:0) | 2.7 |
| LPC(16:0) | 3.2 |
| LPE(14:0) | 8.6 |
| Mannose | 11.6 |
| Methylgingerol | 1.0 |
| MG(14:0) | 1.3 |
| MG(20:5) | 0.6 |
| MG(22:4) | 0.02 |
| MG(22:5) | 1.8 |
| Oxalic acid | 7.3 |
| Palmitic acid | 12.9 |
| Palmitic amide | 5.0 |
| Palmitoylethanolamine | 3.1 |
| PE(15:0/20:1) | 2.7 |
| PG(16:0/18:0) | 2.1 |
| Phosphoric acid | 5.9 |
| Pibutidine | 4.2 |
| PS(18:0/18:0) | 0.3 |
| Pseudouridine | 0.4 |
| Sedanonic acid | 5.7 |
| Sitosterol | 0.4 |
| Sorgolactone | 0.8 |
| Sphinganine | 3.7 |
| Stearic acid | 14.3 |
| Theaspirone A | 2.6 |
| Tocopherol | 14.0 |
| Undecanedioic acid | 2.0 |

Table S3. $Q^2$ and $R^2$ values from PLS-DA of C, C-Se, Abx and Abx-Se groups

| Parameters | $Q^2$ | | | | $R^2Y$ | | | |
|---|---|---|---|---|---|---|---|---|
| Groups | C-Se vs C | Abx vs C | Abx-Se vs C | Abx-Se vs Abx | C-Se vs C | Abx vs C | Abx-Se vs C | Abx-Se vs Abx |
| GC-MS | 0.879 | 0.857 | 0.912 | 0.946 | 0.999 | 0.998 | 0.999 | 1 |
| ESI(+)-UHPLC-QTOF-MS | 0.798 | 0.956 | 0.645 | 0.79 | 0.986 | 0.998 | 0.924 | 0.989 |
| ESI(-)-UHPLC-QTOF-MS | 0.638 | 0.901 | 0.687 | 0.935 | 0.915 | 0.994 | 0.926 | 0.999 |

Table S4. Gut altered metabolites ordered by class. RT: retention time, FC: Fold change, N.S.: Not significant, lysophosphatidylcholine (LPC), lysophosphatidylethanolamine (LPE), phosphatidylethanolamine (PE); phosphatidylglycerol (PG); phosphatidylserine (PS); monoglyceride (MG); *p*-value obtained from one way ANOVA followed by Tuckey Test and corrected by Benjamini-Hochberg multiple post-correction.

| Experimental Mass (Da) | Theorical Mass (Da) | RT (min) | **Compound** | **Abx-Se vs Abx** | | **Abx vs C** | | **Abx-Se vs C** | | **C-Se vs C** | | **Mode** | **Adduct** | **Class** | **Sub Class** |
|---|---|---|---|---|---|---|---|---|---|---|---|---|---|---|---|
| | | | | **p** | **FC** | **p** | **FC** | **p** | **FC** | **p** | **FC** | | | | |
| 308.2008 | 308.1987 | 9.31 | Methylgingerol | N.S | 3.31 | N.S | 1.20 | 0.01 | 3.99 | N.S | 1.81 | ESI- | $[M-H]^{+}$ | Benzene and substituted derivatives | Methoxybenzenes |
| 164.0848 | 164.0837 | 13.11 | 1-(4-Methoxyphenyl)-2-propanone | 0.01 | 0.93 | N.S. | 1.18 | N.S. | 1.09 | N.S. | 2.88 | ESI+ | $[M+H]^{+}$ | Benzene and substituted derivatives | Phenylpropanes |
| 118.0786 | 118.0783 | 8.76 | Methylstyrene | 0.01 | 1.45 | 0.009 | 1.54 | N.S. | 2.23 | N.S. | 1.12 | ESI+ | $[M+H]^{+}$ | Benzene and substituted derivatives | Phenylpropenes |
| 98 | 97.9769 | 16 | Phosphoric acid | 0.001 | 1.29 | N.S | 1.68 | 0.02 | 2.16 | 0.2 | 1.46 | GC-MS | - | Non-metal oxoanionic compounds | Non-metal phosphates |
| 298 | 298.1205 | 25.48 | Enterolactone | 0.01 | 0.10 | 0.002 | 0.23 | 0.0002 | 0.02 | 0.0002 | 0.05 | GC-MS | - | Furanoid lignans | Tetrahydrofuran lignans |
| 286.2146 | 286.2144 | 9.69 | 16-Hydroxy-10-oxohexadecanoic acid | 0.0002 | 0.62 | 0.002 | 1.94 | N.S | 1.20 | N.S | 1.03 | ESI- | $[M-H]^{-}$ | Fatty Acyls | Fatty acids and conjugates |
| 288.1931 | 288.1937 | 4.05 | 6-Hydroxypentadecanedioic acid | 0.003 | 3.00 | 0.0001 | 0.57 | N.S | 1.71 | N.S | 1.05 | ESI- | $[M-H]^{-}$ | Fatty Acyls | Fatty acids and conjugates |
| 310.2154 | 310.2144 | 9.35 | 12,13-Epoxy-11-hydroxy-9,15-octadecadienoic acid | 0.04 | 0.62 | N.S | 3.14 | N.S | 1.93 | N.S | 3.39 | ESI- | $[M-H]^{-}$ | Fatty Acyls | Fatty acids and conjugates |
| 316.2627 | 316.2613 | 8.02 | 9,10-Dihydroxystearic acid | N.S | 1.61 | N.S | 0.17 | 0.05 | 0.27 | 0.001 | 0.27 | ESI- | $[M-H]^{-}$ | Fatty Acyls | Fatty acids and conjugates |
| 382.381 | 382.3810 | 15.28 | 6-Hydroxy-8-pentacosanone | 0.0001 | 0.59 | 0.0001 | 1.26 | N.S | 0.75 | 0.003 | 1.78 | ESI- | $[M-H]^{-}$ | Fatty Acyls | Fatty acids and conjugates |
| 256.2402 | 256 | 19 | Palmitic acid | N.S | 1.54 | 0.007 | 0.59 | N.S | 0.92 | 0.03 | 0.69 | GC-MS | - | Fatty Acyls | Fatty acids and conjugates |
| 284.2715 | 284 | 20.77 | Stearic acid | 0.003 | 2.94 | 0.03 | 0.39 | 0.005 | 0.25 | 0.03 | 0.49 | GC-MS | - | Fatty Acyls | Fatty acids and conjugates |
| 188.1049 | 188 | 16.47 | Azelaic acid | 0.001 | 0.17 | N.S | 1.24 | 0.0002 | 0.22 | 0.0002 | 0.20 | GC-MS | - | Fatty Acyls | Fatty acids and conjugates |
| 216.1359 | 216.1361 | 6.39 | Undecanedioic acid | 0.002 | 0.33 | N.S | 0.80 | 0.02 | 0.27 | 0.04 | 0.29 | ESI- | $[M-H]^{-}$ | Fatty Acyls | Fatty acids and conjugates |
| 294.2203 | 294.2195 | 6.8 | Colneleic acid | 0.0002 | 0.31 | N.S | 1.60 | 0.01 | 0.50 | N.S | 0.61 | ESI- | $[M-H]^{-}$ | Fatty Acyls | Fatty acids and conjugates |
| 398.3768 | 398.3760 | 16.09 | 2-Hydroxy-22-methyltetracosanoic acid | 0.0002 | 1.30 | 0.0002 | 1.61 | N.S | 2.10 | 0.01 | 1.32 | ESI- | $[M-H]^{-}$ | Fatty Acyls | Fatty acids and conjugates |
| 396.3971 | 396.3967 | 15.91 | 6-Hydroxy-8-hexacosanone | 0.0001 | 0.60 | 0.0001 | 1.35 | N.S | 0.81 | 0.004 | 1.26 | ESI- | $[M-H]^{-}$ | Fatty Acyls | Fatty alcohols |
| 356.3625 | 356.3654 | 15.22 | Erythro-6,8-Tricosanediol | 0.0001 | 0.77 | 0.0001 | 2.09 | N.S | 1.61 | 0.003 | 3.42 | ESI- | $[M-H]^{-}$ | Fatty Acyls | Fatty alcohols |

| | | | | | | | | | | | | | | | |
|---|---|---|---|---|---|---|---|---|---|---|---|---|---|---|---|
| 384.3964 | 384.3967 | 16.65 | Erythro-6,8-Pentacosanediol | 0.0001 | 0.68 | 0.0001 | 2.62 | N.S | 1.79 | 0.003 | 3.92 | ESI- | $[M-H]^-$ | Fatty Acyls | Fatty alcohols |
| 255.2556 | 255.2562 | 12.42 | Palmitic amide | 0.01 | 0.74 | N.S. | 0.56 | N.S. | 0.42 | N.S. | 0.56 | ESI+ | $[M+H]^+$ | Fatty Acyls | Fatty amides |
| 554.4910 | 554.4910 | 15.6 | DG(15:0/16:0) | 0.02 | 1.28 | N.S. | 1.21 | N.S. | 1.54 | N.S. | 0.89 | ESI+ | $[M+H]^+$ | Glycerolipids | Diradylglycerols |
| 302.2502 | 302.2457 | 5.46 | MG(14:0) | 0.02 | 1.70 | N.S | 1.20 | N.S | 2.05 | N.S | 0.67 | ESI+ | $[M+H]^+$ | Glycerolipids | Monoradylglycerols |
| 376.2658 | 376.2613 | 10.42 | MG(20:5) | N.S | 1.40 | N.S | 1.41 | 0.04 | 1.97 | N.S | 0.95 | ESI+ | $[M+H]^+$ | Glycerolipids | Monoradylglycerols |
| 404.2969 | 404.2926 | 11.4 | MG(22:5) | 0.01 | 1.17 | N.S | 1.42 | N.S | 1.65 | 0.01 | 0.54 | ESI+ | $[M+H]^+$ | Glycerolipids | Monoradylglycerols |
| 406.312 | 406.3083 | 12.03 | MG(22:4) | 0.004 | 2.59 | 0.01 | 1.02 | N.S | 2.63 | 0.02 | 0.60 | ESI+ | $[M+H]^+$ | Glycerolipids | Monoradylglycerols |
| 260.1263 | 260.1260 | 4.76 | Glycerol tripropanoate | 0.04 | 0.41 | N.S | 1.02 | N.S | 0.41 | N.S | 0.56 | ESI- | $[M-H]^-$ | Glycerolipids | Triradylcglycerols |
| 481.3194 | 481.3246 | 10.95 | LPC(15:0) | N.S. | 1.38 | 0.01 | 1.04 | N.S. | 1.44 | 0.01 | 9.33 | ESI+ | $[M+H]^+$ | Glycerophospholipids | Glycerophosphocholines |
| 495.3325 | 495.3403 | 10.02 | LPC(16:0) | 0.04 | 1.40 | N.S. | 0.87 | N.S. | 1.22 | N.S. | 2.06 | ESI+ | $[M+H]^+$ | Glycerophospholipids | Glycerophosphocholines |
| 425.2556 | 425.2545 | 8.56 | LPE(14:0) | 0.004 | 0.28 | N.S | 1.50 | 0.03 | 0.42 | N.S | 0.58 | ESI- | $[M-H]^-$ | Glycerophospholipids | Glycerophosphoethanola mines |
| 731.5413 | 731.5465 | 11.29 | PE(15:0/20:1) | 0.04 | 0.43 | N.S. | 1.21 | N.S. | 0.52 | N.S. | 0.58 | ESI+ | $[M+H]^+$ | Glycerophospholipids | Glycerophosphoethanola mines |
| 750.5406 | 750.5410 | 15.34 | PG(16:0/18:0) | 0.0001 | 0.36 | 0.0001 | 4.57 | N.S | 1.65 | 0.005 | 1.20 | ESI- | $[M-H]^-$ | Glycerophospholipids | Glycerophosphoglycerol s |
| 791.5627 | 791.5676 | 15.17 | PS(18:0/18:0) | 0.0002 | 1.09 | 0.0002 | 0.76 | N.S | 0.83 | 0.004 | 1.24 | ESI- | $[M-H]^-$ | Glycerophospholipids | Glycerophosphoserines |
| 262.2295 | 262.2296 | 10.24 | 6,10,14-Trimethyl-5,9,13-pentadecatrien-2-one | 0.006 | 0.77 | 0.04 | 0.61 | N.S. | 0.47 | N.S. | 0.45 | ESI+ | $[M+H]^+$ | Prenol lipids | Diterpenoids |
| 416.3651 | 416 | 27.43 | Tocopherol | N.S | 1.22 | 0.004 | 0.49 | N.S | 0.59 | 0.001 | 0.40 | GC-MS | - | Prenol lipids | Quinone and hydroquinone lipids |
| 318.2026 | 318.2042 | 4.98 | L-Citronellol glucoside | N.S | 0.13 | N.S | 1.71 | 0.008 | 0.23 | N.S | 0.54 | ESI- | $[M-H]^-$ | Prenol lipids | Terpene glycosides |
| 316.1334 | 316.1311 | 5.01 | Sorgolactone | N.S | 1.67 | 0.0004 | 0.24 | 0.03 | 0.39 | N.S | 0.48 | ESI- | $[M-H]^-$ | Prenol lipids | Terpene lactones |
| 428.3651 | 428.3654 | 17.53 | 4-Hydroxymethyl-4-methyl-5-cholesta-8,24-dien-3-ol | 0.0004 | 0.16 | N.S. | 0.83 | N.S. | 0.14 | N.S. | 0.12 | ESI+ | $[M+H]^+$ | Prenol lipids | Triterpenoids |
| 376.3019 | 376.2977 | 11.59 | 7-Hydroxy-5-cholanic acid | 0.005 | 2.87 | 0.02 | 0.44 | N.S | 1.26 | N.S | 0.64 | ESI- | $[M-H]^-$ | Steroids and steroid derivatives | Bile acids, alcohols and derivatives |
| 436.3594 | 436.3552 | 11.77 | 5-Cholestane-3,7,12,23-Tetrol | 0.003 | 3.23 | N.S | 0.93 | 0.008 | 3.00 | N.S | 1.82 | ESI- | $[M-H]^-$ | Steroids and steroid derivatives | Bile acids, alcohols and derivatives |
| 386.3549 | 386 | 27.57 | Cholesterol | N.S | 1.09 | N.S | 1.59 | N.S | 1.31 | 0.02 | 1.95 | GC-MS | - | Steroids and steroid derivatives | Cholestane steroids |
| 388.3696 | 388.3705 | 9.69 | Coprostanol | 0.0007 | 1.24 | N.S. | 1.08 | N.S. | 1.34 | N.S. | 1.16 | ESI+ | $[M+H]^+$ | Steroids and steroid derivatives | Cholestane steroids |
| 416.4018 | 416 | 29.33 | 24-Ethylcoprostanol | 0.05 | 0.88 | 0.04 | 3.49 | 0.4 | 1.08 | N.S | 0.58 | GC-MS | - | Steroids and steroid derivatives | Cholestane steroids |

| | | | | | | | | | | | | | | | |
|---|---|---|---|---|---|---|---|---|---|---|---|---|---|---|---|
| 384.3405 | 384.3405 | 17.5 | Desmosterol | 0.009 | 1.14 | N.S. | 1.00 | N.S. | 1.14 | N.S. | 2.54 | ESI+ | $[M+H]^+$ | Steroids and steroid derivatives | Cholestane steroids |
| 400.3705 | 400 | 28.44 | Campesterol | 0.01 | 3.24 | 0.01 | 2.03 | 0.01 | 2.05 | 0.005 | 2.44 | GC-MS | - | Steroids and steroid derivatives | Ergostane steroids |
| 426.3519 | 426.3498 | 13.11 | 6-Hydroxystigmasta-4,22-dien-3-one | 0.04 | 1.10 | N.S. | 0.79 | N.S. | 0.87 | N.S. | 2.35 | ESI+ | $[M+H]^+$ | Steroids and steroid derivatives | Stigmastanes and derivatives |
| 462.3735 | 462.3708 | 13.35 | 6-Deoxohomodolichosterone | 0.002 | 0.94 | N.S. | 0.73 | N.S. | 0.68 | N.S. | 1.40 | ESI+ | $[M+H]^+$ | Steroids and steroid derivatives | Stigmastanes and derivatives |
| 476.3513 | 476.3501 | 12.75 | Homodolichosterone | 0.001 | 2.33 | N.S | 0.28 | N.S | 0.65 | N.S | 0.24 | ESI- | $[M-H]^-$ | Steroids and steroid derivatives | Stigmastanes and derivatives |
| 414.3861 | 414 | 29.22 | Sitosterol | N.S | 1.73 | N.S | 0.71 | N.S | 0.71 | 0.03 | 1.61 | GC-MS | - | Steroids and steroid derivatives | Stigmastanes and derivatives |
| 444.3275 | 444.3239 | 11.12 | 1, 24,25-Trihydroxyvitamin D2 | 0.007 | 2.16 | N.S | 0.88 | 0.02 | 1.91 | N.S | 1.50 | ESI- | $[M-H]^-$ | Steroids and steroid derivatives | Vitamin D and derivatives |
| 244.0700 | 244.0695 | 0.48 | Pseudouridine | N.S | 0.84 | N.S | 0.88 | N.S | 0.74 | 0.005 | 0.56 | ESI- | $[M-H]^-$ | Nucleoside and nucleotide analogues | Nucleosides, nucleotides, and analogues |
| 268.0817 | 268.0808 | 0.36 | Arabinosylhypoxanthine | N.S | 1.43 | 0.007 | 1.89 | 0.02 | 2.70 | 0.003 | 7.33 | ESI- | $[M-H]^-$ | Purine nucleosides | Purine nucleosides |
| 268.0808 | 268 | 23.42 | Inosine | N.S | 0.93 | N.S | 0.83 | N.S | 0.78 | 0.03 | 0.35 | GC-MS | - | Purine nucleosides | Purine nucleosides |
| 299.282 | 299.2824 | 9.32 | Palmitoylethanolamine | 0.05 | 1.26 | N.S. | 0.87 | N.S. | 1.10 | N.S. | 0.94 | ESI+ | $[M+H]^+$ | Carboximidic acids and derivatives | Carboximidic acids |
| 232.1426 | 232.1423 | 1.01 | Isoleucyl-Threonine | 0.03 | 1.03 | N.S | 0.68 | N.S | 0.70 | N.S | 1.04 | ESI- | $[M-H]^-$ | Carboxylic acids and derivatives | Amino acids, peptides, and analogues |
| 294.1063 | 294.1063 | 5.04 | Distichonic acid | 0.03 | 0.59 | N.S | 1.46 | N.S | 0.86 | N.S | 0.86 | ESI- | $[M-H]^-$ | Carboxylic acids and derivatives | Amino acids, peptides, and analogues |
| 188.1159 | 188.1161 | 1.17 | Glycyl-L-leucine | 0.002 | 1.66 | N.S | 0.82 | N.S | 1.36 | N.S | 1.54 | ESI- | $[M-H]^-$ | Carboxylic acids and derivatives | Amino acids, peptides, and analogues |
| 90 | 89.9953 | 7.22 | Oxalic acid | N.S | 1.05 | N.S | 1.22 | 0.04 | 0.72 | N.S | 2.83 | GC-MS | - | Carboxylic acids and derivatives | Dicarboxylic acids and derivatives |
| 146.0947 | 146.0943 | 4.82 | 3-Hydroxyisoheptanoic acid | 0.01 | 0.98 | 0.01 | 1.29 | N.S | 1.26 | N.S | 1.42 | ESI- | $[M-H]^-$ | Hydroxy acids and derivatives | Medium-chain hydroxy acids and derivatives |
| 130.0628 | 130.0629 | 1.97 | Ethyl 3-oxobutanoate | 0.002 | 0.82 | 0.0003 | 2.43 | N.S | 1.98 | N.S | 1.29 | ESI- | $[M-H]^-$ | Keto acids and derivatives | Beta-keto acids and derivatives |
| 210.125 | 210.1256 | 6.28 | Sedanonic acid | N.S. | 0.97 | 0.03 | 0.87 | N.S. | 0.85 | N.S. | 1.32 | ESI+ | $[M+H]^+$ | Keto acids and derivatives | Gamma-keto acids and derivatives |
| 210.093 | 210.093 | 3.58 | 2-Ethyl-1-hexanol sulfate | 0.003 | 0.35 | N.S | 1.52 | N.S | 0.54 | N.S | 0.60 | ESI- | $[M-H]^-$ | Organic sulfuric acids and derivatives | Sulfuric acid esters |
| 301.2974 | 301.2980 | 9.06 | Sphinganine | 0.01 | 1.42 | 0.0003 | 0.30 | N.S. | 0.43 | 0.001 | 1.78 | ESI+ | $[M+H]^+$ | Organonitrogen compounds | Amines |
| 356.185 | 356.1848 | 7.98 | Pibutidine | 0.01 | 1.11 | N.S | 0.87 | N.S | 0.97 | N.S | 0.93 | ESI- | $[M-H]^-$ | Organonitrogen compounds | Amines |
| 380.3638 | 380.3654 | 14.5 | 10,12-Pentacosanedione | 0.0001 | 0.78 | 0.0001 | 1.25 | N.S | 0.97 | 0.003 | 2.07 | ESI- | $[M-H]^-$ | Organooxygen compounds | Carbonyl compounds |

| | | | | | | | | | | | | | | | |
|---|---|---|---|---|---|---|---|---|---|---|---|---|---|---|---|
| 180.0634 | 180 | 21.25 | Mannose | N.S | 1.82 | N.S | 1.93 | 0.01 | 3.51 | 0.05 | 2.13 | GC-MS | - | Organooxygen compounds | Carbohydrates and carbohydrate conjugates |
| 92.0473 | 92 | 16 | Glycerol | N.S | 2.76 | 0.0003 | 0.33 | N.S | 0.96 | 0.0002 | 0.20 | GC-MS | - | Organooxygen compounds | Carbohydrates and carbohydrate conjugates |
| 324.1116 | 324.1143 | 4.68 | Acetohexamide | N.S | 0.72 | N.S | 0.59 | 0.03 | 0.42 | N.S | 1.77 | ESI- | $[M-H]^-$ | Organooxygen compounds | Carbonyl compounds |
| 138.0665 | 138.0681 | 2.46 | 1-(2-Furanyl)-1-butanone | N.S | 0.70 | 0.05 | 4.68 | N.S | 3.29 | N.S | 0.95 | ESI- | $[M-H]^-$ | Organooxygen compounds | Carbonyl compounds |
| 208.148 | 208.1463 | 8.04 | Theaspirone A | N.S | 1.06 | 0.04 | 1.51 | N.S | 1.61 | N.S | 1.38 | ESI- | $[M-H]^-$ | Organooxygen compounds | Carbonyl compounds |
| 366.3846 | 366.3861 | 16.7 | 7-Pentacosanone | 0.0005 | 1.65 | 0.0004 | 1.63 | N.S | 2.69 | N.S | 1.36 | ESI- | $[M-H]^-$ | Organooxygen compounds | Carbonyl compounds |
| 191.0598 | 191.05824 | 3.81 | 5-Hydroxyindoleacetic acid | N.S | 1.08 | N.S | 1.37 | 0.02 | 1.48 | N.S | 0.94 | ESI- | $[M-H]^-$ | Indoles and derivatives | Indolyl carboxylic acids and derivatives |
| 360.2339 | 360.2314 | 9.4 | Calycanthidine | N.S | 0.77 | 0.01 | 0.88 | N.S | 0.68 | N.S | 1.69 | ESI- | $[M-H]^-$ | Indoles and derivatives | Pyrroloindoles |

Table S5. Kovat´s retention Index (KRI) of GC-MS metabolites.

| **Compound name** | Exact Mass (Da) | MW (Da) | Derivatives | MW-Derivatives (Da) | RT (min) | KRI | Target ion (m/z) | Qualifier ions (m/z) |
|---|---|---|---|---|---|---|---|---|
| Oxalic acid | 89.9953 | 90 | 2-TMS | 234 | 7.22 | 1126 | 190 | 147, 175, 219 |
| Glycerol | 92.0473 | 92 | 3-TMS | 308 | 10 | 1263 | 205 | 218, 293 |
| Phosphoric acid | 97.9769 | 98 | 3-TMS | 315 | 10.1 | 1277 | 315 | 299, 256 |
| Azelaic acid | 188.1049 | 188 | 2-TMS | 332 | 16.47 | 1675 | 317 | 129, 201,217 |
| Mannose | 180.0634 | 180 | 1-MOX, 5-TMS | 540 | 16.77 | 1804 | 319 | 204, 217, 191 |
| Palmitic acid | 256.2402 | 256 | 1-TMS | 328 | 19.01 | 2050 | 313 | 201, 269 |
| Stearic acid | 284.2715 | 284 | 1-TMS | 356 | 20.77 | 2125 | 341 | 359, 297 |
| Inosine | 268.0808 | 268 | 4-TMS | 556 | 23.42 | 2583 | 217 | 230, 217, 281 |
| Enterolactone | 298.1205 | 298 | 2-TMS | 442 | 25.48 | 2843 | 180 | 205, 217, 442 |
| Tocopherol | 416.3651 | 416 | 1-TMS | 502 | 27.43 | 3145 | 502 | 237, 277 |
| Cholesterol | 386.3549 | 386 | 1-TMS | 458 | 27.57 | 3150 | 329 | 368, 353, 368 |
| Campesterol | 400.3705 | 400 | 1-TMS | 472 | 28.44 | 3166 | 343 | 382, 367, 472 |
| Sitosterol | 414.3861 | 414 | 1-TMS | 486 | 29.22 | 3198 | 396 | 357, 486 |
| 24-Ethylcoprostanol | 416.4018 | 416 | 1-TMS | 488 | 29.33 | 3300 | 215 | 383, 473 |

MW: Molecular Weight; TMS: Trimethylsilyl derivatives, MOX: Methoxyamine derivative.

Table S6. Pathway analysis details of altered metabolites in Abx and Abx-Se groups. Match Status: number of altered metabolites of the total metabolites involving in the route, p-value p value calculated from the enrichment analysis; Impact: pathway impact value calculated from pathway topology analysis.

| **Pathway Name** | **Match Status** | **p** | **Impact** |
|---|---|---|---|
| Phenylalanine, tyrosine and tryptophan biosynthesis | 1/4 | 0.04 | 0.5 |
| Biosynthesis of unsaturated fatty acids | 2/36 | 0.06 | 0.0 |
| Steroid biosynthesis | 2/42 | 0.08 | 0.03 |
| Ubiquinone and other terpenoid-quinone biosynthesis | 1/9 | 0.09 | 0.0 |
| Phenylalanine metabolism | 1/12 | 0.13 | 0.0 |
| Glycerolipid metabolism | 1/16 | 0.17 | 0.2 |
| Sphingolipid metabolism | 1/21 | 0.21 | 0.2 |
| Galactose metabolism | 1/27 | 0.27 | 0.0 |
| Glycerophospholipid metabolism | 1/36 | 0.34 | 0.02 |
| Fatty acid elongation | 1/39 | 0.36 | 0.0 |
| Fatty acid degradation | 1/39 | 0.36 | 0.0 |
| Tyrosine metabolism | 1/42 | 0.38 | 0.1 |
| Primary bile acid biosynthesis | 1/46 | 0.41 | 0.03 |
| Fatty acid biosynthesis | 1/47 | 0.42 | 0.02 |
| Aminoacyl-tRNA biosynthesis | 1/48 | 0.43 | 0.0 |
| Purine metabolism | 1/66 | 0.54 | 0.002 |
| Steroid hormone biosynthesis | 1/77 | 0.59 | 0.006 |

Table S7. Taxa Abundance at genus level in C, C-Se, Abx and Abx-Se groups

| | Taxa | C | C-Se | Abx | Abx-Se |
|---|---|---|---|---|---|
| *1)* | *Acetatifactor* | 0.370 | 0.857 | 0.637 | 0.166 |
| *2)* | *Acinetobacter* | 0.003 | 0.074 | 0.017 | 0.016 |
| *3)* | *Akkermansia* | 0.014 | 0.017 | 0.018 | 0.019 |
| *4)* | *Alistipes* | 1.887 | 1.487 | 0.484 | 1.390 |
| *5)* | *Alloprevotella* | 1.627 | 0.424 | 0.276 | 0.097 |
| *6)* | *Anaerofustis* | 0.008 | 0.032 | 0.001 | 0.008 |
| *7)* | *Anaeroplasma* | 0.002 | 0.093 | 0.002 | 0.116 |
| *8)* | *Anaerotruncus* | 0.552 | 0.856 | 0.992 | 0.685 |
| *9)* | *Angelakisella* | 0.032 | 0.072 | 0.062 | 0.049 |
| *10)* | *Bacteroides* | 1.439 | 3.102 | 7.313 | 2.795 |
| *11)* | *Bilophila* | 0.280 | 0.462 | 0.344 | 0.047 |
| *12)* | *Butyricicoccus* | 0.557 | 0.545 | 0.090 | 0.302 |
| *13)* | *Candidatus_Saccharimonas* | 0.173 | 0.133 | 0.000 | 0.000 |
| *14)* | *Candidatus_Stoquefichus* | 0.013 | 0.009 | 0.023 | 0.000 |
| *15)* | *Caulobacter* | 0.004 | 0.011 | 0.030 | 0.012 |
| *16)* | *Desulfovibrio* | 1.202 | 0.413 | 0.001 | 0.000 |
| *17)* | *Eggerthellaceae_DNF00809* | 0.339 | 0.549 | 0.317 | 0.443 |
| *18)* | *Enterorhabdus* | 0.526 | 0.702 | 0.726 | 0.857 |
| *19)* | *Erysipelatoclostridium* | 0.001 | 0.006 | 0.160 | 0.565 |
| *20)* | *EscherichiaShigella* | 0.028 | 0.044 | 0.049 | 0.053 |
| *21)* | *Faecalibacterium* | 0.011 | 0.018 | 0.022 | 0.015 |
| *22)* | *Family_XIII_UCG001* | 0.170 | 0.190 | 0.143 | 0.179 |
| *23)* | *Flavonifractor* | 0.241 | 0.187 | 0.069 | 0.044 |
| *24)* | *Harryflintia* | 0.003 | 0.039 | 0.000 | 0.011 |

| | | | | | |
|---|---|---|---|---|---|
| 25) | *Intestinimonas* | 0.289 | 0.575 | 0.304 | 0.590 |
| 26) | *Lachnoclostridium* | 6.911 | 10.34 | 8.324 | 11.883 |
| 27) | *Lachnospiraceae_A2* | 0.320 | 1.552 | 11.05 | 6.589 |
| 28) | *Lachnospiraceae_ASF356* | 0.007 | 0.016 | 0.001 | 0.017 |
| 29) | *Lachnospiraceae_FCS020_group* | 0.057 | 0.100 | 0.057 | 0.108 |
| 30) | *Lachnospiraceae_GCA900066575* | 0.303 | 0.482 | 0.586 | 0.207 |
| 31) | *Lachnospiraceae_NK4A136_group* | 34.25 | 18.42 | 24.51 | 21.36 |
| 32) | *Lachnospiraceae_UCG001* | 2.587 | 2.006 | 1.535 | 0.742 |
| 33) | *Lachnospiraceae_UCG004* | 0.116 | 0.154 | 0.050 | 0.176 |
| 34) | *Lachnospiraceae_UCG006* | 3.230 | 3.921 | 1.698 | 2.389 |
| 35) | *Lactobacillus* | 2.142 | 2.133 | 0.008 | 1.721 |
| 36) | *Marvinbryantia* | 0.006 | 0.177 | 0.029 | 0.130 |
| 37) | *Mucispirillum* | 0.413 | 0.964 | 0.037 | 0.077 |
| 38) | *Muribaculum* | 0.192 | 0.481 | 0.478 | 0.168 |
| 39) | *Odoribacter* | 1.717 | 1.115 | 0.095 | 0.152 |
| 40) | *Oscillibacter* | 1.124 | 1.395 | 1.261 | 0.804 |
| 41) | *Parabacteroides* | 0.062 | 0.137 | 1.394 | 0.060 |
| 42) | *Parvibacter* | 0.096 | 0.026 | 0.017 | 0.021 |
| 43) | *Peptococcus* | 0.037 | 0.069 | 0.022 | 0.006 |
| 44) | *Prevotellaceae_UCG001* | 0.301 | 0.166 | 0.063 | 0.015 |
| 45) | *Rikenellaceae_RC9_gut_group* | 1.030 | 0.194 | 0.359 | 0.318 |
| 46) | *Roseburia* | 2.996 | 3.653 | 4.819 | 4.862 |
| 47) | *Ruminiclostridium* | 0.246 | 0.562 | 0.081 | 0.074 |
| 48) | *Ruminiclostridium_5* | 0.376 | 1.068 | 0.264 | 0.498 |
| 49) | *Ruminiclostridium_6* | 0.712 | 0.280 | 0.342 | 0.177 |
| 50) | *Ruminiclostridium_9* | 0.901 | 0.917 | 1.580 | 0.759 |
| 51) | *Ruminococcaceae_GCA900066225* | 0.094 | 0.149 | 0.001 | 0.000 |

| | | | | | |
|---|---|---|---|---|---|
| *52)* | *Ruminococcaceae_NK4A214_group* | 0.041 | 0.033 | 0.014 | 0.017 |
| *53)* | *Ruminococcaceae_UCG003* | 0.283 | 0.074 | 0.075 | 0.004 |
| *54)* | *Ruminococcaceae_UCG005* | 0.086 | 0.029 | 0.010 | 0.001 |
| *55)* | *Ruminococcaceae_UCG009* | 0.027 | 0.052 | 0.007 | 0.002 |
| *56)* | *Ruminococcaceae_UCG010* | 0.096 | 0.090 | 0.002 | 0.026 |
| *57)* | *Ruminococcaceae_UCG014* | 1.033 | 0.542 | 0.052 | 0.715 |
| *58)* | *Ruminococcus_1* | 0.560 | 0.207 | 0.048 | 0.141 |
| *59)* | *Staphylococcus* | 0.021 | 0.059 | 0.004 | 0.006 |
| *60)* | *Streptococcus* | 0.316 | 0.668 | 0.210 | 0.485 |
| *61)* | *Subdoligranulum* | 0.009 | 0.021 | 0.020 | 0.024 |

Table S8. Spearman correlation coefficient (rho) of altered gut metabolites and genus. Lysophosphatidylcholine (LPC), lysophosphatidylethanolamine (LPE), phosphatidylethanolamine (PE); phosphatidylglycerol (PG); phosphatidylserine (PS); monoglyceride (MG)

| *Genus* | Compound | C | | C-Se | | Abx | | Abx-Se | |
|---|---|---|---|---|---|---|---|---|---|
| | | rho | p | rho | p | rho | p | rho | p |
| *Acetatifactor* | 1,24,25-Trihydroxyvitamin D2 | | | | | | | 0.81 | 0.027 |
| | 2-Ethyl-1-hexanol sulfate | | | | | | | 0.93 | 0.008 |
| | 2-Hydroxy-22-methyltetracosanoic acid | | | | | 0.82 | 0.02 | | |
| | Calycanthidine | -0.83 | 0.04 | | | | | | |
| | Campesterol | | | | | | | -0.78 | 0.023 |
| | Distichonic acid | | | | | | | 0.79 | 0.02 |
| | MG(22:5) | -0.89 | 0.02 | | | | | | |
| | Palmitic acid | | | | | | | 0.79 | 0.02 |
| | 12,13-Epoxy-11-hydroxy-9,15-octadecadienoic acid | | | | | | | -0.75 | 0.031 |
| *Acinetobacter* | 1,24,25-Trihydroxyvitamin D2 | | | | | | | -0.9 | 0.006 |
| | 2-Ethyl-1-hexanol sulfate | | | | | | | -0.83 | 0.039 |
| | 2-Hydroxy-22-methyltetracosanoic acid | | | | | 0.8 | 0.03 | | |
| | 7-Pentacosanone | | | | | 0.8 | 0.03 | | |
| | Erythro-6,8-Tricosanediol | | | -0.94 | 0.01 | | | | |
| | L-Citronellol glucoside | | | | | | | -0.77 | 0.025 |
| | LysoPC(16:0) | | | -0.79 | 0.02 | | | | |
| | Pseudouridine | | | | | | | -0.9 | 0.006 |
| *Akkermansia* | 1-(2-Furanyl)-1-butanone | | | | | -0.92 | 0 | | |
| | 1-(4-Methoxyphenyl)-2-propanone | | | | | 0.8 | 0.02 | | |
| | 16-Hydroxy-10-oxohexadecanoic acid | | | 0.83 | 0.01 | | | | |
| | 6-Hydroxy-8-pentacosanone | | | | | 0.8 | 0.03 | | |
| | Methylstyrene | | | | | 0.82 | 0.02 | | |

| | | | | | | | | | |
|---|---|---|---|---|---|---|---|---|---|
| | Azelaic acid | | | | | | | -0.86 | 0.007 |
| | Glycerol tripropanoate | | | | | -0.92 | 0 | | |
| | LPC(15:0) | | | | | 0.93 | 0 | | |
| | Palmitic amide | | | -0.83 | 0.04 | | | | |
| | Undecanedioic acid | 0.84 | 0.02 | | | | | | |
| *Alistipes* | 4-Methyl-5-cholesta-8,24-dien-3-one | 0.83 | 0.04 | | | | | | |
| | 5-Cholestane-3,7,12,23-Tetrol | | | | | 0.79 | 0.03 | | |
| | 6,10,14-Trimethyl-5,9,13-pentadecatrien-2-one | | | | | 0.78 | 0.04 | | |
| | 6-Hydroxy-8-hexacosanone | | | | | -0.81 | 0.03 | | |
| | 6-Hydroxy-8-pentacosanone | | | | | -0.77 | 0.04 | | |
| | 7-Hydroxy-5-cholanic acid | | | | | 0.79 | 0.03 | | |
| | Azelaic acid | | | | | 0.92 | 0 | | |
| | Campesterol | | | | | | | -0.86 | 0.007 |
| | Distichonic acid | | | | | 0.77 | 0.04 | | |
| | Erythro-6,8-Pentacosanediol | | | -0.83 | 0.04 | | | | |
| | Erythro-6,8-Tricosanediol | | | | | -0.95 | 0 | | |
| | LPC(15:0) | 0.83 | 0.04 | | | | | | |
| | LPC(16:0) | | | | | | | -0.9 | 0.037 |
| | MG(22:5) | -0.83 | 0.04 | | | | | | |
| | Oxalic acid | | | -0.77 | 0.02 | | | | |
| | Pseudouridine | | | | | 0.83 | 0.02 | | |
| | Sphinganine | 0.83 | 0.04 | | | | | | |
| | Stearic acid | 0.77 | 0.02 | | | | | | |
| | Tocopherol | 0.8 | 0.01 | | | | | | |
| | 12,13-Epoxy-11-hydroxy-9,15-octadecadienoic acid | | | | | | | -0.76 | 0.028 |
| *Alloprevotella* | Methylstyrene | 0.9 | 0.04 | | | | | | |
| | Azelaic acid | | | 0.85 | 0 | | | | |

| | | | | | | | | | |
|---|---|---|---|---|---|---|---|---|---|
| | Undecanedioic acid | -0.86 | 0.01 | | | | | | |
| *Anaerofustis* | 2-Ethyl-1-hexanol sulfate | | | -0.82 | 0.02 | | | | |
| | Inositol | | | | | | | -0.76 | 0.027 |
| | Stearic acid | | | -0.82 | 0.01 | | | | |
| *Anaeroplasma* | 24-Ethylcoprostanol | | | | | | | 0.79 | 0.02 |
| | 2-Ethyl-1-hexanol sulfate | | | | | 0.79 | 0.03 | | |
| | 4-Methyl-5-cholesta-8,24-dien-3-one | | | | | 0.76 | 0.03 | | |
| | Methylstyrene | | | -0.89 | 0.01 | | | | |
| | L-Citronellol glucoside | | | | | 0.79 | 0.03 | | |
| | LPC(16:0) | | | | | | | -0.9 | 0.037 |
| | Pseudouridine | | | | | 0.79 | 0.03 | | |
| | Sorgolactone | | | -0.8 | 0.01 | | | | |
| | Undecanedioic acid | | | | | 0.79 | 0.03 | | |
| *Anaerotruncus* | 6-Hydroxystigmasta-4,22-dien-3-one | | | | | -0.83 | 0.01 | | |
| | 24-Ethylcoprostanol | | | | | | | -0.86 | 0.007 |
| | 6-Deoxohomodolichosterone | | | | | -0.96 | 0 | | |
| | Methylstyrene | | | | | -0.93 | 0 | | |
| | Campesterol | 0.82 | 0.01 | | | | | | |
| | L-Citronellol glucoside | | | | | 0.82 | 0.02 | -0.76 | 0.028 |
| | Theaspirone A | 0.86 | 0.01 | | | | | -0.86 | 0.014 |
| | Erythro-6,8-Pentacosanediol | | | -0.83 | 0.04 | | | | |
| *Angelakisella* | 6-Hydroxystigmasta-4,22-dien-3-one | | | 0.88 | 0 | | | | |
| | 1-(4-Methoxyphenyl)-2-propanone | | | 0.86 | 0.01 | | | | |
| | 5-Hydroxyindoleacetic acid | -0.89 | 0 | | | -0.79 | 0.04 | | |
| | 6-Deoxohomodolichosterone | | | 0.82 | 0.01 | | | | |
| | Arabinosylhypoxanthine | | | | | -0.89 | 0.04 | | |
| | Campesterol | | | | | | | -0.85 | 0.007 |

| | | | | | | | | | |
|---|---|---|---|---|---|---|---|---|---|
| | Colneleic acid | | | | | -0.79 | 0.04 | | |
| | DG(15:0/16:0) | | | | | -0.76 | 0.03 | 0.83 | 0.042 |
| | LPC(15:0) | | | 0.76 | 0.03 | | | | |
| | Sphinganine | | | 0.9 | 0 | | | | |
| | 12,13-Epoxy-11-hydroxy-9,15-octadecadienoic acid | | | | | | | -0.76 | 0.03 |
| *Bacteroides* | 1-(2-Furanyl)-1-butanone | | | | | 0.89 | 0.01 | | |
| | 16-hydroxy-10-oxo-hexadecanoic acid | | | -0.92 | 0 | | | 0.79 | 0.021 |
| | 4-Methyl-5-cholesta-8,24-dien-3-one | | | 0.89 | 0.02 | | | 0.82 | 0.023 |
| | Colneleic acid | | | -0.83 | 0.01 | | | | |
| | Glycerol tripropanoate | | | | | 0.79 | 0.04 | | |
| | LPC(15:0) | 0.89 | 0.02 | | | | | | |
| | MG(14:0) | | | -0.9 | 0.04 | | | | |
| | MG(20:5) | | | | | -0.79 | 0.04 | | |
| | Palmitic amide | | | 0.83 | 0.04 | | | | |
| | Sphinganine | 0.89 | 0.02 | | | | | | |
| | Theaspirone A | | | | | | | 0.89 | 0.007 |
| | Tocopherol | 0.75 | 0.02 | | | | | | |
| *Bilophila* | 4-Hydroxymethyl-4-methyl-5-cholesta-8,24-dien-3b-ol | | | | | 0.81 | 0.02 | | |
| | 4-Methyl-5-cholesta-8,24-dien-3-one | 0.89 | 0.02 | | | | | | |
| | 5-Hydroxyindoleacetic acid | -0.78 | 0.02 | | | | | | |
| | LPC(15:0) | 0.89 | 0.02 | | | | | | |
| | Sedanonic acid | | | | | 0.85 | 0.01 | | |
| | Sphinganine | 0.89 | 0.02 | 0.79 | 0.02 | | | | |
| | Stearic acid | | | | | | | 0.76 | 0.027 |
| *Butyricicoccus* | 2-Ethyl-1-hexanol sulfate | | | | | | | -0.87 | 0.024 |
| | DG(15:0/16:0) | | | 0.76 | 0.03 | | | | |
| | Erythro-6,8-Pentacosanediol | | | 0.89 | 0.02 | | | | |

| | | | | | | | | | |
|---|---|---|---|---|---|---|---|---|---|
| | LPC(16:0) | | | | | | | 0.9 | 0.037 |
| | Methylgingerol | | | | | -0.79 | 0.04 | | |
| | Palmitic acid | | | | | | | -0.83 | 0.011 |
| *Candidatus_Saccharimonas* | 6-Deoxohomodolichosterone | -0.89 | 0.02 | | | | | | |
| | Colneleic acid | -0.79 | 0.02 | | | | | | |
| | Glycerol tripropanoate | 0.9 | 0 | | | | | | |
| | Methylgingerol | | | -0.83 | 0.01 | | | | |
| | Phosphoric acid | | | -0.75 | 0.02 | | | | |
| | Theaspirone A | -0.79 | 0.04 | | | | | | |
| *Candidatus_Stoquefichus* | Erythro-6,8-Pentacosanediol | | | | | -0.82 | 0.02 | | |
| | Mannose | | | | | 0.9 | 0 | | |
| | Sphinganine | | | | | 0.8 | 0.02 | | |
| *Caulobacter* | 4-Hydroxymethyl-4-methyl-5-cholesta-8,24-dien-3b-ol | | | | | -0.79 | 0.02 | | |
| | 6-Hydroxy-8-hexacosanone | | | | | 0.82 | 0.02 | | |
| | Azelaic acid | | | -0.76 | 0.02 | | | | |
| | DG(15:0/16:0) | | | | | | | 0.91 | 0.011 |
| | Distichonic acid | | | | | -0.82 | 0.02 | | |
| | L-Citronellol glucoside | | | | | -0.8 | 0.03 | | |
| | MG(14:0) | | | 0.89 | 0.04 | | | | |
| | Pseudouridine | | | | | -0.78 | 0.04 | | |
| | Sedanonic acid | | | | | -0.78 | 0.04 | | |
| | Undecanedioic acid | | | 0.85 | 0.03 | | | | |
| | 12,13-Epoxy-11-hydroxy-9,15-octadecadienoic acid | | | | | | | -0.75 | 0.031 |
| *Desulfovibrio* | Arabinosylhypoxanthine | | | -0.85 | 0.03 | | | | |
| | Calycanthidine | 0.85 | 0.03 | | | | | | |
| | MG(22:5) | 0.85 | 0.03 | | | | | | |
| *Eggerthellaceae_DNF00809* | 24-Ethylcoprostanol | -0.8 | 0.01 | | | | | | |

| | | | | | | | | | |
|---|---|---|---|---|---|---|---|---|---|
| | 2-Ethyl-1-hexanol sulfate | -0.77 | 0.04 | | | | | | |
| | 2-Hydroxy-22-methyltetracosanoic acid | | | | | -0.79 | 0.04 | | |
| | L-Citronellol glucoside | -0.77 | 0.03 | | | | | | |
| *Enterorhabdus* | Methylstyrene | -0.9 | 0.04 | | | | | | |
| | L-Citronellol glucoside | -0.83 | 0.01 | | | | | | |
| | MG(20:5) | | | | | | | -0.83 | 0.01 |
| | Palmitic amide | 0.89 | 0.02 | | | | | | |
| *Erysipelatoclostridium* | 1,24,25-Trihydroxyvitamin D2 | | | | | | | -0.77 | 0.041 |
| | Arabinosylhypoxanthine | | | | | 0.97 | 0 | | |
| | DG(15:0/16:0) | | | | | | | -0.94 | 0.005 |
| | Distichonic acid | | | | | | | -0.75 | 0.031 |
| | Enterolactone | | | 0.84 | 0 | | | | |
| | Homodolichosterone | | | | | -0.77 | 0.04 | | |
| | L-Citronellol glucoside | | | 0.76 | 0.02 | | | -0.78 | 0.023 |
| | MG(20:5) | | | 0.78 | 0.01 | | | | |
| | Palmitoylethanolamine | | | -0.93 | 0.01 | | | | |
| | Pseudouridine | | | | | | | -0.88 | 0.008 |
| | Theaspirone A | | | 0.76 | 0.03 | | | | |
| *EscherichiaShigella* | 1-(2-Furanyl)-1-butanone | | | | | -0.85 | 0.01 | | |
| | 16-hydroxy.10-oxo-hexadecanoic acid | | | 0.83 | 0.01 | | | | |
| | 6-Deoxohomodolichosterone | | | | | 0.77 | 0.04 | | |
| | Methylstyrene | | | | | 0.77 | 0.04 | | |
| | Glycerol tripropanoate | | | | | -0.78 | 0.04 | | |
| | LysoPC(15:0) | | | | | 0.79 | 0.02 | | |
| | Sedanonic acid | | | | | -0.81 | 0.03 | | |
| *Faecalibacterium* | alpha-Methylstyrene | | | 0.8 | 0.03 | | | | |
| | Azelaic acid | | | -0.81 | 0.01 | | | | |

| | | | | | | | | | |
|---|---|---|---|---|---|---|---|---|---|
| | Colneleic acid | | | | | 0.77 | 0.04 | | |
| | Desmosterol | | | 0.79 | 0.02 | | | | |
| | Enterolactone | -0.77 | 0.01 | | | | | | |
| | L-Citronellol glucoside | 0.87 | 0.01 | | | | | | |
| | MG(20:5) | | | | | 0.84 | 0.02 | | |
| | 9,10-dihydroxyoctadecanoic acid | | | | | | | 0.75 | 0.031 |
| | 24-Ethylcoprostanol | | | | | | | -0.79 | 0.02 |
| | 4-Hydroxymethyl-4-methyl-5-cholesta-8,24-dien-3-ol | | | | | 0.95 | 0 | | |
| | 6-Hydroxy-8-hexacosanone | | | | | -0.81 | 0.03 | | |
| | Distichonic acid | | | | | 0.85 | 0.02 | | |
| *Family_XIII_UCG001* | Glycerol tripropanoate | | | | | 0.77 | 0.04 | | |
| | L-Citronellol glucoside | | | | | 0.83 | 0.02 | | |
| | Mannose | 0.79 | 0.01 | | | | | | |
| | MG(22:5) | | | 0.9 | 0 | | | | |
| | Sedanonic acid | | | | | 0.82 | 0.03 | | |
| | Theaspirone A | 0.89 | 0.01 | | | | | | |
| | 9,10-dihydroxyoctadecanoic acid | | | | | | | 0.75 | 0.031 |
| | 1,24,25-Trihydroxyvitamin D2 | | | | | | | 0.89 | 0.007 |
| | 4-Hydroxymethyl-4-methyl-5-cholesta-8,24-dien-3-ol | | | | | 0.84 | 0.01 | | |
| | 4-Methyl-5-cholesta-8,24-dien-3-one | | | | | 0.77 | 0.03 | | |
| | 5-Cholestane-3,7,12,23-Tetrol | | | | | | | 0.9 | 0.002 |
| *Flavonifractor* | 5-Hydroxyindoleacetic acid | | | | | | | 0.8 | 0.017 |
| | 7-Hydroxy-5-cholanic acid | | | | | | | 0.85 | 0.007 |
| | Glycerol | | | | | | | 0.97 | 0 |
| | MG(20:5) | -0.81 | 0.01 | | | | | 0.9 | 0.002 |
| | MG(22:5) | | | | | | | 0.92 | 0.001 |
| | Pseudouridine | | | | | | | 0.79 | 0.036 |

| | | | | | | | | | |
|---|---|---|---|---|---|---|---|---|---|
| *Harryflintia* | 6-Hydroxystigmasta-4,22-dien-3-one | 0.88 | 0.02 | | | | | | |
| | 6,10,14-Trimethyl-5,9,13-pentadecatrien-2-one | | | | | | | 0.89 | 0.041 |
| | Methylstyrene | | | -0.85 | 0.02 | | | | |
| | MG(22:4) | -0.88 | 0.02 | | | | | | |
| | Palmitic amide | 0.88 | 0.02 | | | | | | |
| | Stearic acid | 0.82 | 0.01 | | | | | | |
| | Tocopherol | 0.82 | 0.01 | | | | | | |
| *Intestinimonas* | 1-(4-Methoxyphenyl)-2-propanone | | | | | | | -0.83 | 0.042 |
| | 1,24,25-Trihydroxyvitamin D2 | | | | | | | 0.93 | 0.003 |
| | 2-Ethyl-1-hexanol sulfate | | | | | | | 0.94 | 0.005 |
| | 4-Hydroxymethyl-4-methyl-5-cholesta-8,24-dien-3-ol | -0.89 | 0.02 | | | | | | |
| | Coprostanol | | | | | 0.79 | 0.02 | | |
| | Campesterol | | | | | | | -0.79 | 0.021 |
| | Cholesterol | | | | | -0.79 | 0.01 | | |
| | Desmosterol | | | | | 0.86 | 0.01 | | |
| | Distichonic acid | | | | | | | 0.83 | 0.01 |
| | Glycyl-L-leucine | | | | | 0.83 | 0.04 | | |
| | MG(20:5) | | | | | | | 0.76 | 0.028 |
| | Palmitic amide | | | | | -0.76 | 0.03 | | |
| | Pseudouridine | | | | | | | 0.82 | 0.023 |
| | Undecanedioic acid | | | -0.83 | 0.04 | | | | |
| *Lachnoclostridium* | 24-Ethylcoprostanol | | | | | | | 0.88 | 0.004 |
| | 6,10,14-Trimethyl-5,9,13-pentadecatrien-2-one | | | | | | | -0.9 | 0.037 |
| | Inosine | 0.8 | 0.01 | | | | | | |
| | Isoleucyl-Threonine | | | | | -0.9 | 0.04 | | |
| | L-Citronellol glucoside | | | | | | | 0.79 | 0.021 |
| | Sphinganine | | | -0.81 | 0.01 | | | | |

| | | | | | | | | | |
|---|---|---|---|---|---|---|---|---|---|
| | Theaspirone A | | | | | | | 0.93 | 0.003 |
| *Lachnospiraceae_A2* | 1,24,25-Trihydroxyvitamin D2 | | | | | | | -0.79 | 0.036 |
| | 2-Ethyl-1-hexanol sulfate | | | | | | | -0.83 | 0.042 |
| | 4-Hydroxymethyl-4-methyl-5-cholesta-8,24-dien-3-ol | | | | | -0.95 | 0 | | |
| | 5-Cholestane-3,7,12,23-Tetrol | -0.83 | 0.01 | | | | | | |
| | 6-Deoxohomodolichosterone | 0.83 | 0.04 | | | | | | |
| | Methylstyrene | | | -0.82 | 0.02 | | | | |
| | Distichonic acid | | | | | | | -0.83 | 0.01 |
| | Pseudouridine | | | | | | | -0.79 | 0.036 |
| *Lachnospiraceae_ASF356* | 1,24,25-Trihydroxyvitamin D2 | | | | | | | -0.8 | 0.03 |
| | 5-Hydroxyindoleacetic acid | | | | | | | -0.85 | 0.008 |
| | 6-Hydroxy-8-hexacosanone | | | 0.85 | 0.03 | | | | |
| | Erythro-6,8-Tricosanediol | | | 0.85 | 0.03 | | | | |
| | Glycerol | | | | | | | -0.76 | 0.027 |
| | Pseudouridine | | | | | | | -0.8 | 0.03 |
| *Lachnospiraceae_FCS020_group* | 1-(2-Furanyl)-1-butanone | | | | | 0.77 | 0.04 | | |
| | 24-Ethylcoprostanol | | | -0.83 | 0.01 | | | | |
| | 3-Hydroxyisoheptanoic acid | | | | | 0.9 | 0.04 | | |
| | 5-Cholestane-3,7,12,23-Tetrol | -0.88 | 0 | | | | | | |
| | 6-Hydroxy-8-pentacosanone | | | | | -0.9 | 0.01 | | |
| | Distichonic acid | | | | | 0.79 | 0.03 | | |
| | Glycerol tripropanoate | -0.76 | 0.03 | | | 0.86 | 0.01 | | |
| | Sedanonic acid | | | | | 0.85 | 0.01 | | |
| *Lachnospiraceae_GCA900066576* | 9,10-dihydroxyoctadecanoic acid | | | | | 0.89 | 0.01 | | |
| | 6-Deoxohomodolichosterone | 0.89 | 0.02 | | | | | | |
| | Glycerol tripropanoate | -0.9 | 0 | | | | | | |
| | Sphinganine | | | | | 0.76 | 0.03 | | |

| | | | | | | | | | |
|---|---|---|---|---|---|---|---|---|---|
| | Theaspirone A | 0.79 | 0.04 | | | | | | |
| *Lachnospiraceae_NK4A136_group* | 6-Hydroxystigmasta-4,22-dien-3-one | | | | | -0.81 | 0.01 | | |
| *Lachnospiraceae_NK4A136_group* | 4-Methyl-5-cholesta-8,24-dien-3-one | -0.89 | 0.02 | | | | | | |
| | 5-Hydroxyindoleacetic acid | 0.81 | 0.01 | | | | | | |
| | 6,10,14-Trimethyl-5,9,13-pentadecatrien-2-one | | | | | | | 0.9 | 0.037 |
| | 6-Deoxohomodolichosterone | | | | | -0.82 | 0.02 | | |
| | Arabinosylhypoxanthine | | | | | -0.9 | 0.04 | | |
| | Enterolactone | | | | | -0.84 | 0 | | |
| | Erythro-6,8-Tricosanediol | | | 0.94 | 0 | | | | |
| | Glycyl-L-leucine | | | | | 0.83 | 0.04 | | |
| | LPC(15:0) | -0.89 | 0.02 | | | | | | |
| | PS(18:0/18:0) | | | 0.83 | 0.04 | | | | |
| | Sphinganine | -0.89 | 0.02 | | | | | | |
| *Lachnospiraceae_UCG001* | 1,24,25-Trihydroxyvitamin D2 | | | | | | | 0.82 | 0.023 |
| | 6-Deoxohomodolichosterone | 0.83 | 0.04 | | | | | | |
| | Homodolichosterone | | | | | -0.86 | 0.01 | | |
| | Inosine | | | | | | | 0.9 | 0.002 |
| | L-Citronellol glucoside | -0.92 | 0 | | | | | | |
| | LysoPE(0:0/14:0) | | | | | | | 0.76 | 0.028 |
| | Mannose | | | -0.87 | 0 | | | | |
| | PS(18:0/18:0) | | | 0.83 | 0.04 | | | | |
| | Sitosterol | -0.78 | 0.01 | | | | | | |
| | 12,13-Epoxy-11-hydroxy-9,15-octadecadienoic acid | | | -0.78 | 0.01 | | | | |
| *Lachnospiraceae_UCG004* | 6-Hydroxystigmasta-4,22-dien-3-one | 0.84 | 0.04 | | | | | | |
| | 24-Ethylcoprostanol | | | | | | | 0.83 | 0.01 |
| | 2-Ethyl-1-hexanol sulfate | | | | | -0.85 | 0.01 | | |
| | 6,10,14-Trimethyl-5,9,13-pentadecatrien-2-one | | | | | | | -0.9 | 0.037 |

| | | | | | | | | | |
|---|---|---|---|---|---|---|---|---|---|
| | Arabinosylhypoxanthine | | | | | -0.89 | 0.04 | -0.9 | 0.037 |
| | Homodolichosterone | | | | | 0.78 | 0.04 | | |
| | Inosine | 0.82 | 0.01 | | | | | | |
| | MG(14:0) | | | 0.9 | 0.04 | | | | |
| | Palmitic amide | 0.84 | 0.04 | | | | | | |
| | Palmitoylethanolamine | | | | | -0.76 | 0.03 | | |
| | Sphinganine | | | -0.76 | 0.03 | | | | |
| | Theaspirone A | | | | | | | 0.79 | 0.036 |
| | Undecanedioic acid | | | | | -0.85 | 0.01 | | |
| *Lachnospiraceae_UCG006* | 2-Hydroxy-22-methyltetracosanoic acid | | | -0.83 | 0.04 | | | | |
| | 6-Hydroxy-8-pentacosanone | | | -0.89 | 0.02 | | | | |
| | 7-Pentacosanone | | | -0.83 | 0.04 | | | | |
| | Arabinosylhypoxanthine | | | 0.83 | 0.04 | | | | |
| | Glycerol tripropanoate | | | 0.82 | 0.01 | | | | |
| | LPC(15:0) | | | -0.76 | 0.03 | | | | |
| | LPC(16:0) | | | -0.83 | 0.01 | | | | |
| | MG(20:5) | | | 0.85 | 0 | | | | |
| | MG(20:5) | | | 0.87 | 0 | | | | |
| | Palmitic acid | | | | | | | -0.76 | 0.028 |
| | Palmitoylethanolamine | | | | | | | 0.9 | 0.037 |
| | Palmitoylethanolamine | | | -0.89 | 0.02 | | | | |
| | Tocopherol | | | | | | | 0.81 | 0.015 |
| *Lactobacillus* | 5-Cholestane-3,7,12,23-Tetrol | -0.81 | 0.01 | | | | | | |
| | 6,10,14-Trimethyl-5,9,13-pentadecatrien-2-one | | | | | | | 0.9 | 0.037 |
| | 6-Deoxohomodolichosterone | 0.83 | 0.04 | | | | | | |
| | Azelaic acid | | | | | | | -0.89 | 0.003 |
| | Campesterol | | | | | | | 0.89 | 0.003 |

| | | | | | | | | | |
|---|---|---|---|---|---|---|---|---|---|
| | Glycerol tripropanoate | -0.79 | 0.02 | | | | | | |
| | L-Citronellol glucoside | | | 0.75 | 0.02 | | | | |
| | Palmitic acid | | | | | | | -0.75 | 0.031 |
| | PG(16:0/18:0) | | | 0.83 | 0.04 | | | | |
| *Marvinbryantia* | 10,12-Pentacosanedione | | | | | 0.79 | 0.03 | | |
| | 1,24,25-Trihydroxyvitamin D2 | | | | | | | 0.77 | 0.041 |
| | 5-Cholestane-3,7,12,23-Tetrol | -0.87 | 0.01 | | | | | | |
| | 6-Deoxohomodolichosterone | 0.93 | 0.01 | | | | | | |
| | 6-Hydroxy-8-hexacosanone | | | | | 0.85 | 0.02 | | |
| | 6-Hydroxy-8-pentacosanone | | | | | 0.94 | 0 | | |
| | Methylstyrene | | | -0.87 | 0.01 | | | | |
| | Distichonic acid | | | | | -0.88 | 0.01 | | |
| | Pseudouridine | | | | | -0.83 | 0.02 | 0.95 | 0.001 |
| *Mucispirillum* | 6-Hydroxystigmasta-4,22-dien-3-one | | | | | | | -0.85 | 0.034 |
| | 24-Ethylcoprostanol | | | | | | | 0.76 | 0.027 |
| | 2-Ethyl-1-hexanol sulfate | | | 0.79 | 0.04 | | | | |
| | 4-Methyl-5-cholesta-8,24-dien-3-one | 0.89 | 0.02 | | | | | | |
| | L-Citronellol glucoside | | | | | | | 0.76 | 0.027 |
| | LPC(15:0) | 0.89 | 0.02 | | | | | | |
| | LPE(14:0) | | | 0.8 | 0.01 | | | | |
| | MG(22:5) | -0.94 | 0 | | | | | | |
| | Palmitic amide | | | | | | | -0.85 | 0.034 |
| | Sphinganine | 0.89 | 0.02 | | | | | | |
| | Stearic acid | 0.76 | 0.02 | | | | | | |
| | Tocopherol | 0.76 | 0.02 | | | | | | |
| *Muribaculum* | 1-(4-Methoxyphenyl)-2-propanone | 0.83 | 0.04 | | | | | | |
| | 2-Ethyl-1-hexanol sulfate | -0.82 | 0.02 | | | | | | |

| | | | | | | | | | |
|---|---|---|---|---|---|---|---|---|---|
| | 4-Hydroxymethyl-4-methyl-5-cholesta-8,24-dien-3b-ol | 0.94 | 0 | | | | | | |
| | 4-Methyl-5-cholesta-8,24-dien-3-one | 0.83 | 0.04 | | | | | | |
| | 5-Hydroxyindoleacetic acid | | | 0.75 | 0.02 | | | | |
| | 6-Hydroxypentadecanedioic acid | | | | | | | -0.97 | 0.005 |
| | Campesterol | | | | | | | 0.81 | 0.016 |
| | Enterolactone | 0.81 | 0.01 | | | | | | |
| | LPC(15:0) | 0.83 | 0.04 | | | | | | |
| | Phosphoric acid | | | | | -0.75 | 0.01 | | |
| | Sedanonic acid | | | | | 0.77 | 0.04 | | |
| | Sphinganine | 0.83 | 0.04 | | | | | | |
| | Stearic acid | | | | | | | 0.78 | 0.022 |
| | 16-hydroxy-10-oxohexadecanoic acid | | | -0.8 | 0.01 | | | | |
| | 4-Methyl-5-cholesta-8,24-dien-3-one | 0.89 | 0.02 | | | | | | |
| | Homodolichosterone | | | | | -0.8 | 0.03 | | |
| *Odoribacter* | LPC(15:0) | 0.89 | 0.02 | | | | | | |
| | Methylgingerol | | | | | 0.8 | 0.03 | | |
| | Palmitoylethanolamine | | | 0.89 | 0.02 | | | | |
| | Sphinganine | 0.89 | 0.02 | | | | | | |
| | 1,24,25-Trihydroxyvitamin D2 | | | | | | | 0.82 | 0.023 |
| | 2-Ethyl-1-hexanol sulfate | | | | | | | 0.89 | 0.019 |
| | 4-Hydroxymethyl-4-methyl-5a-cholesta-8,24-dien-3-ol | | | | | 0.93 | 0 | | |
| | 5b-Cholestane-3a,7a,12a,23-Tetrol | -0.83 | 0.01 | | | | | | |
| *Oscillibacter* | Methylstyrene | | | -0.82 | 0.02 | | | | |
| | Campesterol | | | | | | | -0.76 | 0.028 |
| | Distichonic acid | | | | | | | 0.76 | 0.028 |
| | MG(14:0) | | | -0.9 | 0.04 | | | | |
| | Palmitic acid | | | | | | | 0.83 | 0.01 |

| | | | | | | | | | |
|---|---|---|---|---|---|---|---|---|---|
| | 1-(2-Furanyl)-1-butanone | | | 0.75 | 0.02 | | | | |
| | 1-(4-Methoxyphenyl)-2-propanone | | | | | | | -0.88 | 0.021 |
| | 1,24,25-Trihydroxyvitamin D2 | | | | | | | 0.85 | 0.015 |
| | 2-Ethyl-1-hexanol sulfate | | | 0.81 | 0.03 | | | | |
| | 5-Hydroxyindoleacetic acid | | | | | | | 0.76 | 0.03 |
| *Parabacteroides* | 6-Deoxohomodolichosterone | | | | | -0.79 | 0.04 | | |
| | Methylstyrene | | | -0.76 | 0.05 | | | | |
| | Desmosterol | | | | | | | -0.88 | 0.021 |
| | L-Citronellol glucoside | | | | | 0.86 | 0.01 | | |
| | MG(20:5) | | | | | | | 0.85 | 0.007 |
| | Pseudouridine | | | | | | | 0.96 | 0 |
| | 1-(2-Furanyl)-1-butanone | | | | | -0.8 | 0.03 | | |
| | 6-Deoxohomodolichosterone | | | | | 0.8 | 0.03 | | |
| | Methylstyrene | | | | | 0.8 | 0.03 | | |
| | DG(15:0/16:0) | | | | | 0.76 | 0.03 | | |
| *Parvibacter* | Glycerol tripropanoate | | | | | -0.8 | 0.03 | | |
| | Homodolichosterone | | | 0.83 | 0.01 | | | | |
| | L-Citronellol glucoside | -0.9 | 0 | | | | | | |
| | LysoPC(15:0) | | | | | 0.76 | 0.03 | | |
| | Phosphoric acid | | | | | 0.79 | 0.01 | | |
| | 1-(4-Methoxyphenyl)-2-propanone | | | | | | | -0.83 | 0.042 |
| | 4-Methyl-5-cholesta-8,24-dien-3-one | 0.93 | 0.01 | | | | | | |
| | 5-Cholestane-3,7,12,23-Tetrol | | | | | -0.8 | 0.03 | | |
| *Peptococcus* | 5-Hydroxyindoleacetic acid | -0.77 | 0.03 | | | -0.8 | 0.03 | | |
| | 6,10,14-Trimethyl-5,9,13-pentadecatrien-2-one | | | -0.76 | 0.05 | | | | |
| | Arabinosylhypoxanthine | | | | | -0.89 | 0.04 | | |
| | Colneleic acid | | | | | -0.8 | 0.03 | | |

| | | | | | | | | | |
|---|---|---|---|---|---|---|---|---|---|
| | Desmosterol | | | | | | | -0.83 | 0.042 |
| | Erythro-6,8-Pentacosanediol | | | -0.89 | 0.02 | | | | |
| | Erythro-6,8-Tricosanediol | | | | | 0.8 | 0.03 | | |
| | LPC(15:0) | 0.93 | 0.01 | | | | | | |
| | MG(22:5) | | | | | -0.8 | 0.03 | | |
| | Pseudouridine | -0.76 | 0.05 | | | | | | |
| | Sitosterol | | | | | | | 0.76 | 0.03 |
| | Sphinganine | 0.93 | 0.01 | | | | | | |
| | Stearic acid | | | | | | | 0.76 | 0.03 |
| | Theaspirone A | | | | | -0.8 | 0.03 | | |
| *Prevotellaceae_UCG001* | 2-Ethyl-1-hexanol sulfate | -0.86 | 0.01 | | | | | | |
| | 2-Hydroxy-22-methyltetracosanoic acid | | | 0.93 | 0.01 | | | | |
| | 7-Pentacosanone | | | 0.93 | 0.01 | | | | |
| | Calycanthidine | -0.89 | 0.02 | | | | | | |
| | Enterolactone | 0.83 | 0.01 | | | | | | |
| | Erythro-6,8-Tricosanediol | | | 0.93 | 0.01 | | | | |
| | Palmitoylethanolamine | | | 0.93 | 0.01 | | | | |
| | Undecanedioic acid | | | -0.88 | 0.02 | | | | |
| *Rikenellaceae_RC9_gut_group* | 4-Methyl-5-cholesta-8,24-dien-3-one | 0.89 | 0.02 | | | | | | |
| | LPC(15:0) | 0.89 | 0.02 | | | | | | |
| | MG(22:5) | -0.94 | 0 | | | | | | |
| | PS(18:0/18:0) | | | -0.83 | 0.04 | | | | |
| | Sphinganine | 0.89 | 0.02 | | | | | | |
| *Roseburia* | 1-(4-Methoxyphenyl)-2-propanone | | | | | | | -0.94 | 0.005 |
| | 1,24,25-Trihydroxyvitamin D2 | | | | | | | 0.89 | 0.007 |
| | Campesterol | | | | | 0.89 | 0 | -0.79 | 0.021 |
| | Distichonic acid | | | | | | | 0.76 | 0.028 |

| | | | | | | | | | |
|---|---|---|---|---|---|---|---|---|---|
| | Erythro-6,8-Tricosanediol | | | -0.94 | 0 | | | | |
| | MG(22:5) | -0.83 | 0.04 | | | | | | |
| | Pseudouridine | | | | | | | 0.79 | 0.036 |
| *Ruminiclostridium* | 2-Ethyl-1-hexanol sulfate | | | | | 0.77 | 0.04 | | |
| | 4-Methyl-5-cholesta-8,24-dien-3-one | | | | | 0.84 | 0.01 | | |
| | L-Citronellol glucoside | | | | | 0.77 | 0.04 | | |
| | MG(20:5) | -0.79 | 0.02 | | | | | | |
| | Phosphoric acid | 0.77 | 0.02 | | | | | | |
| | Pseudouridine | | | | | | | 0.78 | 0.039 |
| | Undecanedioic acid | -0.82 | 0.02 | | | 0.77 | 0.04 | | |
| *Ruminiclostridium_5* | 6,10,14-Trimethyl-5,9,13-pentadecatrien-2-one | 0.9 | 0.04 | | | | | | |
| | 7-Hydroxy-5-cholanic acid | -0.76 | 0.03 | | | | | | |
| | Colneleic acid | | | | | -0.82 | 0.02 | | |
| | Distichonic acid | | | | | | | 0.81 | 0.015 |
| | MG(20:5) | | | | | -0.89 | 0.01 | 0.81 | 0.015 |
| *Ruminiclostridium_6* | 1-(2-Furanyl)-1-butanone | | | | | 0.82 | 0.03 | | |
| | 24-Ethylcoprostanol | | | | | -0.8 | 0.01 | | |
| | Methylstyrene | -0.9 | 0.04 | | | | | | |
| | Colneleic acid | | | | | -0.82 | 0.03 | | |
| | MG(20:5) | | | | | -0.85 | 0.01 | | |
| | Phosphoric acid | | | | | | | -0.75 | 0.031 |
| | Stearic acid | | | | | | | -0.79 | 0.02 |
| | Undecanedioic acid | 0.86 | 0.01 | | | | | | |
| *Ruminiclostridium_9* | 4-Hydroxymethyl-4-methyl-5a-cholesta-8,24-dien-3-ol | 0.83 | 0.04 | | | 0.88 | 0 | | |
| | Coprostanol | | | | | | | 0.94 | 0.005 |
| | 6-Hydroxy-8-hexacosanone | | | | | -0.86 | 0.01 | | |
| | 6-Hydroxy-8-pentacosanone | | | | | -0.82 | 0.02 | | |

| | | | | | | | | | |
|---|---|---|---|---|---|---|---|---|---|
| | 7-Hydroxy-5-cholanic acid | | | | | 0.79 | 0.04 | | |
| | Campesterol | | | | | | | -0.76 | 0.028 |
| | DG(15:0/16:0) | | | | | | | 0.94 | 0.005 |
| | Distichonic acid | | | | | 0.82 | 0.02 | | |
| | Glycerol tripropanoate | | | | | 0.79 | 0.04 | | |
| | L-Citronellol glucoside | -0.81 | 0.01 | | | | | | |
| | MG(14:0) | | | -0.9 | 0.04 | | | 0.93 | 0.001 |
| | MG(22:5) | -0.89 | 0.02 | | | | | | |
| | Palmitic acid | | | | | | | -0.9 | 0.037 |
| *Ruminococcaceae_GCA900066225* | 4-Hydroxymethyl-4-methyl-5-cholesta-8,24-dien-3-ol | -0.84 | 0.04 | | | | | | |
| | Methylstyrene | | | -0.76 | 0.05 | | | | |
| | Campesterol | -0.77 | 0.02 | | | | | | |
| | Erythro-6,8-Pentacosanediol | | | -0.89 | 0.02 | | | | |
| | Stearic acid | | | 0.82 | 0.01 | | | | |
| *Ruminococcaceae_NK4A214_group* | 1-(4-Methoxyphenyl)-2-propanone | | | | | | | -0.85 | 0.034 |
| | 1,24,25-Trihydroxyvitamin D2 | | | | | | | 0.84 | 0.019 |
| | 2-Ethyl-1-hexanol sulfate | | | | | | | 0.93 | 0.008 |
| | 5-Cholestane-3,7,12,23-Tetrol | | | | | -0.8 | 0.03 | | |
| | 5-Hydroxyindoleacetic acid | | | | | -0.8 | 0.03 | | |
| | Arabinosylhypoxanthine | | | | | -0.89 | 0.04 | | |
| | Colneleic acid | | | | | -0.8 | 0.03 | | |
| | Desmosterol | | | | | | | -0.85 | 0.034 |
| | Erythro-6,8-Tricosanediol | | | | | 0.8 | 0.03 | | |
| | Inosine | | | -0.82 | 0.01 | | | | |
| | L-Citronellol glucoside | -0.88 | 0 | | | | | | |
| | MG(20:5) | | | | | | | 0.75 | 0.031 |
| | MG(22:5) | | | | | -0.8 | 0.03 | | |

| | | | | | | | | | |
|---|---|---|---|---|---|---|---|---|---|
| | Palmitic amide | 0.9 | 0.01 | | | | | | |
| | Pseudouridine | | | | | | | 0.78 | 0.04 |
| | Stearic acid | 0.83 | 0.01 | | | | | | |
| | Theaspirone A | | | | | -0.8 | 0.03 | | |
| *Ruminococcaceae_UCG003* | 5-Cholestane-3,7,12,23-Tetrol | | | | | -0.87 | 0.01 | | |
| | 7-Hydroxy-5-cholanic acid | | | 0.81 | 0.01 | | | | |
| | Colneleic acid | -0.84 | 0.01 | | | -0.77 | 0.04 | | |
| | Erythro-6,8-Pentacosanediol | | | -0.82 | 0.05 | | | | |
| | Glycerol tripropanoate | | | | | | | 0.76 | 0.03 |
| | MG(20:5) | | | | | -0.81 | 0.03 | | |
| | MG(22:5) | | | | | -0.77 | 0.04 | | |
| | Oxalic acid | | | -0.86 | 0 | | | | |
| | Stearic acid | | | | | | | -0.76 | 0.03 |
| *Ruminococcaceae_UCG005* | 5-Cholestane-3,7,12,23-Tetrol | | | | | -0.84 | 0.02 | | |
| | MG(20:5) | | | | | -0.78 | 0.04 | | |
| | Sorgolactone | -0.76 | 0.03 | | | | | | |
| | Tocopherol | | | 0.92 | 0 | | | | |
| *Ruminococcaceae_UCG009* | 1-(4-Methoxyphenyl)-2-propanone | 0.83 | 0.04 | | | | | | |
| | 2-Ethyl-1-hexanol sulfate | -0.78 | 0.04 | | | | | | |
| | 6,10,14-Trimethyl-5,9,13-pentadecatrien-2-one | -0.9 | 0.04 | | | | | | |
| | Inosine | -0.84 | 0.01 | | | | | | |
| | LPE(14:0) | | | | | -0.8 | 0.03 | | |
| | MG(20:5) | | | | | -0.8 | 0.03 | | |
| *Ruminococcaceae_UCG010* | 5-Hydroxyindoleacetic acid | | | | | | | -0.79 | 0.021 |
| | MG(20:5) | | | | | | | -0.94 | 0.001 |
| | MG(22:5) | | | | | | | -0.91 | 0.005 |
| | Palmitic amide | | | 0.83 | 0.04 | | | | |

| | | | | | | | | | |
|---|---|---|---|---|---|---|---|---|---|
| | Tocopherol | | | | | | | 0.79 | 0.021 |
| | Undecanedioic acid | | | -0.94 | 0 | | | | |
| *Ruminococcaceae_UCG014* | 1,24,25-Trihydroxyvitamin D2 | | | | | | | 0.82 | 0.023 |
| | 2-Ethyl-1-hexanol sulfate | | | | | | | 0.83 | 0.042 |
| | 4-Methyl-5-cholesta-8,24-dien-3-one | 0.94 | 0 | | | | | | |
| | 5-Hydroxyindoleacetic acid | -0.9 | 0 | | | | | | |
| | Distichonic acid A | -0.75 | 0.03 | | | | | | |
| | LPC(15:0) | 0.94 | 0 | | | | | | |
| | MG(20:5) | | | | | -0.79 | 0.03 | | |
| | Pseudouridine | | | | | | | 0.93 | 0.003 |
| | Sorgolactone | | | -0.79 | 0.01 | | | | |
| | Sphinganine | 0.94 | 0 | | | | | | |
| *Ruminococcus_1* | 6-Hydroxystigmasta-4,22-dien-3-one | | | | | 0.76 | 0.03 | | |
| | 1-(4-Methoxyphenyl)-2-propanone | 0.94 | 0 | | | | | | |
| | 2-Ethyl-1-hexanol sulfate | -0.79 | 0.04 | | | | | | |
| | 4-Hydroxymethyl-4-methyl-5-cholesta-8,24-dien-3-ol | 0.83 | 0.04 | | | | | | |
| | Coprostanol | 0.89 | 0.02 | | | | | | |
| | 6,10,14-Trimethyl-5,9,13-pentadecatrien-2-one | -0.9 | 0.04 | | | | | | |
| | 6-Deoxohomodolichosterone | | | | | 0.79 | 0.04 | | |
| | Methylstyrene | | | | | 0.79 | 0.04 | | |
| | Desmosterol | 0.89 | 0.02 | | | | | | |
| | Enterolactone | 0.81 | 0.01 | | | | | | |
| | Glycerol tripropanoate | | | | | -0.79 | 0.04 | | |
| | Glycyl-L-leucine | | | | | -0.88 | 0.02 | | |
| | L-Citronellol glucoside | | | | | -0.87 | 0.01 | | |
| | Mannose | 0.88 | 0 | | | | | | |
| | MG(14:0) | | | -0.89 | 0.04 | | | | |

| | | | | | | | | | |
|---|---|---|---|---|---|---|---|---|---|
| | Pseudouridine | | | | | | | 0.78 | 0.04 |
| | Sorgolactone | | | | | 0.91 | 0 | | |
| *Staphylococcus* | 9,10-dihydroxyoctadecanoic acid | | | | | | | -0.79 | 0.019 |
| | 1-(4-Methoxyphenyl)-2-propanone | 0.88 | 0.02 | | | | | | |
| | 4-Hydroxymethyl-4-methyl-5-cholesta-8,24-dien-3-ol | 0.88 | 0.02 | | | | | | |
| | Coprostanol | 0.88 | 0.02 | | | | | | |
| | Campesterol | 0.75 | 0.02 | | | | | | |
| | Desmosterol | 0.88 | 0.02 | | | | | | |
| | Erythro-6,8-Pentacosanediol | | | -0.93 | 0.01 | | | | |
| *Streptococcus* | 16-hydroxy-10-oxo-hexadecanoic acid | | | 0.75 | 0.02 | | | | |
| | 2-Hydroxy-22-methyltetracosanoic acid | | | | | -0.79 | 0.04 | | |
| | Coprostanol | -0.82 | 0.05 | | | | | | |
| | 6,10,14-Trimethyl-5,9,13-pentadecatrien-2-one | 0.97 | 0 | | | | | | |
| | Colneleic acid | | | 0.77 | 0.02 | | | | |
| | Desmosterol | -0.82 | 0.05 | | | | | | |
| | Erythro-6,8-Pentacosanediol | | | | | 0.79 | 0.04 | | |
| | MG(14:0) | | | 0.9 | 0.04 | | | | |
| | Palmitic acid | | | | | -0.78 | 0.01 | | |
| | Palmitic amide | | | -0.83 | 0.04 | | | | |
| | Sphinganine | | | | | -0.76 | 0.03 | | |
| *Subdoligranulum* | 1-(2-Furanyl)-1-butanone | | | | | -0.87 | 0.01 | | |
| *Subdoligranulum* | 16-hydroxy-10-oxo-hexadecanoic acid | | | 0.76 | 0.02 | | | | |
| | 4a-Hydroxymethyl-4b-methyl-5a-cholesta-8,24-dien-3b-ol | | | | | -0.79 | 0.02 | | |
| | Calycanthidine | 0.85 | 0.03 | | | | | | |
| | Glycerol tripropanoate | | | | | -0.87 | 0.01 | | |
| | MG(14:0) | | | 0.9 | 0.04 | | | | |
| | MG(22:5) | 0.85 | 0.03 | | | | | | |

| | | | | | | | | | |
|---|---|---|---|---|---|---|---|---|---|
| | Sedanonic acid | | | | | -0.8 | 0.03 | | |
| | Stearic acid | -0.83 | 0.01 | | | | | | |
| | Tocopherol | -0.84 | 0 | | | | | | |
